# Proteomic Comparison of Bone Marrow Derived Osteoblasts and Mesenchymal Stem Cells

**DOI:** 10.3390/ijms22115665

**Published:** 2021-05-26

**Authors:** Elise Aasebø, Annette K. Brenner, Maria Hernandez-Valladares, Even Birkeland, Frode S. Berven, Frode Selheim, Øystein Bruserud

**Affiliations:** 1 Department of Clinical Science, University of Bergen, 5020 Bergen, Norway; elise.aasebo@uib.no (E.A.); annette.brenner@uib.no (A.K.B.); 2 Proteomics Facility of the University of Bergen (PROBE), University of Bergen, 5020 Bergen, Norway; maria.hernandez-valladares@uib.no (M.H.-V.); even.birkeland@uib.no (E.B.); frode.berven@uib.no (F.S.B.); frode.selheim@uib.no (F.S.); 3 Department of Medicine, Haukeland University Hospital, 5021 Bergen, Norway

**Keywords:** bone marrow, osteoblast, mesenchymal stem cell, proteome, in vitro culture, ex vivo handling

## Abstract

Mesenchymal stem cells (MSCs) can differentiate into osteoblasts, and therapeutic targeting of these cells is considered both for malignant and non-malignant diseases. We analyzed global proteomic profiles for osteoblasts derived from ten and MSCs from six healthy individuals, and we quantified 5465 proteins for the osteoblasts and 5420 proteins for the MSCs. There was a large overlap in the profiles for the two cell types; 156 proteins were quantified only in osteoblasts and 111 proteins only for the MSCs. The osteoblast-specific proteins included several extracellular matrix proteins and a network including 27 proteins that influence intracellular signaling (Wnt/Notch/Bone morphogenic protein pathways) and bone mineralization. The osteoblasts and MSCs showed only minor age- and sex-dependent proteomic differences. Finally, the osteoblast and MSC proteomic profiles were altered by ex vivo culture in serum-free media. We conclude that although the proteomic profiles of osteoblasts and MSCs show many similarities, we identified several osteoblast-specific extracellular matrix proteins and an osteoblast-specific intracellular signaling network. Therapeutic targeting of these proteins will possibly have minor effects on MSCs. Furthermore, the use of ex vivo cultured osteoblasts/MSCs in clinical medicine will require careful standardization of the ex vivo handling of the cells.

## 1. Introduction

Various bone marrow stromal cells are important for normal bone formation and remodeling, but they also support both normal and leukemic hematopoiesis as well as metastatic growth of distant cancer [1,2,3,4,5,6,7,8]. Hematopoietic cells have a hierarchical organization, and the minor subsets of normal or leukemic stem cells within these hierarchical populations localize to specialized stem cell niches formed by bone marrow stromal cells [3,4,5,6]. The niches have traditionally been divided into two main types, the osteoblastic or endosteal and the vascular or sinusoidal niche. These two microenvironments seem to have different functions with regard to regulation of stem cell homeostasis, i.e., quiescence and maintenance versus proliferation/differentiation, respectively [3]. A third arteriolar niche has recently been described where periarteriolar stromal cells are important; this niche also seems to maintain quiescence [7]. Osteoblasts and mesenchymal stem/stromal cells (MSCs) are members of these stem cell niches. Furthermore, the extracellular matrix is also important for regulation of hematopoiesis through their effects on communication between various bone marrow cells and regulation of hematopoietic cell localization [1,3]. Thus, bone marrow stromal cells are important both through their cell–cell contacts, extracellular release of specific soluble mediators/proteins (e.g., growth and/or differentiation factors), and formation of the extracellular matrix [8].

Even though osteoblasts differentiate from MSCs [9,10,11], experimental studies suggest that these two cell types differ in their molecular mechanisms for support of normal hematopoiesis [3]. Furthermore, both MSCs and osteoblasts can support carcinogenesis and development of cancer chemoresistance, including homing of circulating cancer cells to the bone marrow with later development of metastases as well as the development of myeloid and lymphoid malignancies [2,3,4,5,6,7,8,9,10,11,12,13,14,15,16,17,18,19]. Targeting of this protective bone marrow microenvironment and the stroma-cancer cell interactions (e.g., blockade by specific monoclonal antibodies) should therefore be considered as a possible therapeutic strategy [19]. Thus, targeting of osteoblasts and/or MSCs is now considered a possible strategy in the treatment of malignant disorders but also nonmalignant bone diseases, e.g., osteoporosis. In this context we have characterized and compared the proteomic profiles of human bone marrow MSCs and osteoblasts.

## 2. Results

### 2.1. Global Proteomic Profiles of Osteoblasts and MSCs

We investigated osteoblasts (10 donors five men and five women; median age 62.5 years with range 53–73 years) and MSCs (six donors; four men and two women; median age 62.5 years with range 47–70 years) derived from healthy individuals. All cell populations were prepared according to the same standardized procedures. Although the cell populations showed differences in their growth characteristics and the time to reach 70% confluence during the first culture period in serum-supplemented medium (see Material and methods Section 4.1), regular light microscopy during and at the end of the culture period (i.e., after 48 h in serum-free medium) showed a large dominance of viable, adherent cells in all the cultures.

More than 4000 proteins were quantified for all MSC and osteoblast populations included in the study. The total number of proteins quantified for each of the two cell types was comparable; we quantified 5465 proteins in osteoblasts and 5420 proteins in the MSCs (see Supplementary file 2 for raw and processed data).

**Figure 1:**
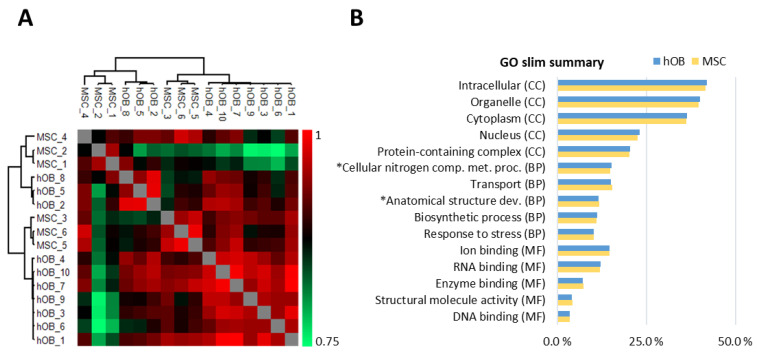
Assessment of the global MSC and osteoblast proteomes. (**A**) Protein intensity correlation analysis based on all samples. The analysis included proteins that showed detectable levels for at least five osteoblast and three MSC donors (Pearson R > 0.76). (**B**) GO slim summary based on the global proteomes of osteoblasts and MSCs. The data were obtained from a GO tool, and the top five annotated GO terms reflecting cellular compartments (CC), biological processes (BP) and molecular functions (MF) are presented. * The full GO terms are “cellular nitrogen compound metabolic process” and “anatomical structure development”.

Global proteome analyses were based on proteins quantified in at least 50% of the samples for each of the two cell types; 4749 proteins were detected for at least five osteoblast donors and 4875 proteins for at least three MSC donors. Protein intensity correlation analysis based on all samples showed a strong correlation (Pearson R > 0.76), and the correlations between donor samples were particularly strong when comparing the same cell type. For the ten osteoblast samples the average Pearson R value was 0.92 (range 0.85–0.96), and for the six MSC samples it was 0.89 (range 0.82–0.95) (Figure 1A). Gene ontology (GO) analysis of the osteoblast and MSC proteomes showed that the cells contained comparable percentages of proteins in GO terms that reflect main biological processes, cellular compartments, and molecular functions (Figure 1B).

### 2.2. Qualitative Proteomic Differences between Osteoblasts and MSCs: A Minor Protein Subset Is Only Detected in Osteoblasts and Reflects Their Differentiation and/or Specialized Functions

Osteoblasts differentiate from MSCs [9,10,11], and to further characterize this differentiation process we first characterized the proteins that reached detectable levels only for osteoblasts but not for any of the six MSC cell populations. A total of 156 proteins were quantified only for osteoblasts, i.e., were detected for at least one osteoblast donor, and the complete list of these proteins is presented in Table S1. The osteoblast-expressed proteins that reflect the differentiation status or the specialized functions of these cells are listed in Table 1, and more detailed descriptions of their biological characteristics are presented in Table S2. Osteoblasts were especially characterized by expression of several extracellular matrix proteins or proteins involved in the organization of the extracellular matrix, but these cells also expressed several proteins that are important for ossification. Furthermore, Notch, Wnt, and integrin signaling is important for osteoblast differentiation (Table S2) [20], and osteoblasts expressed several proteins that are important for these pathways. However, bone morphogenic proteins (BMPs) are also important for osteoblast differentiation and regulation of specialized osteoblastic functions [21,22], but none of the proteins only detected in osteoblasts are known to be involved in BMP signaling (GO:0030509 BMP signaling pathway, GO:0030510 regulation of BMP signaling pathway) (data not shown).

We did a subclassification of the osteoblast-specific proteins based on GO enrichment using a GO tool (Table 2), and the results showed that the terms cell membrane and signal were overrepresented among osteoblast-specific proteins, while nuclear proteins were underrepresented. The proteins also differed in their biological functions.

**Figure 2:**
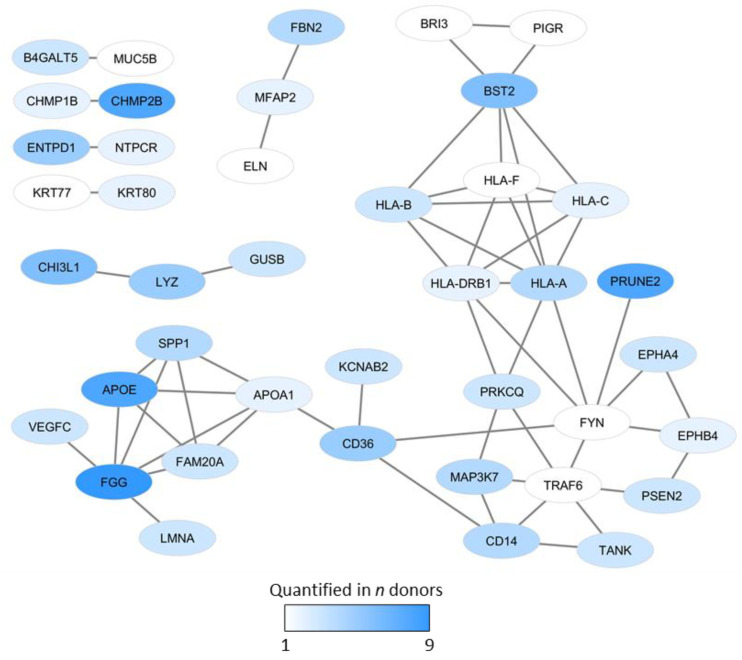
Osteoblast-specific protein interaction networks. The figure presents networks from the String database based on proteins only reaching quantifiable levels in osteoblasts; 41 of the 156 osteoblast-specific proteins were connected in the various networks. The color key gradient from white to blue indicates the number of donors with quantifiable levels for each of the proteins (e.g., only one donor white color, at least nine donors the darkest blue).

We finally did a network analysis of the osteoblast-specific proteins, and 41 of the 156 proteins were connected in various networks (Figure 2). Furthermore, the more detailed description of the network proteins given in Table S3 shows that most of these proteins are important or characteristic for osteoblastic differentiation and/or reflect (the regulation of) important functional characteristics of osteoblasts.

### 2.3. A Minor Subset of Diverse Proteins Reach Quantifiable Levels Only for MSCs

A minor subset of 111 proteins were quantified only for MSCs (Table S4). Analysis of GO terms based on these 111 proteins could not identify any significant terms (data not shown), and relatively few of these proteins were included in protein interactions (Figure S1).

### 2.4. Quantitative Proteomic Differences between Osteoblasts and MSCs: Proteins Showing Increased Levels in Osteoblasts Reflect Cellular Differentiation and/or Specialized Functions whereas Proteins Increased in MSCs Reflect Differences in Transcriptional Regulation

**Figure 3:**
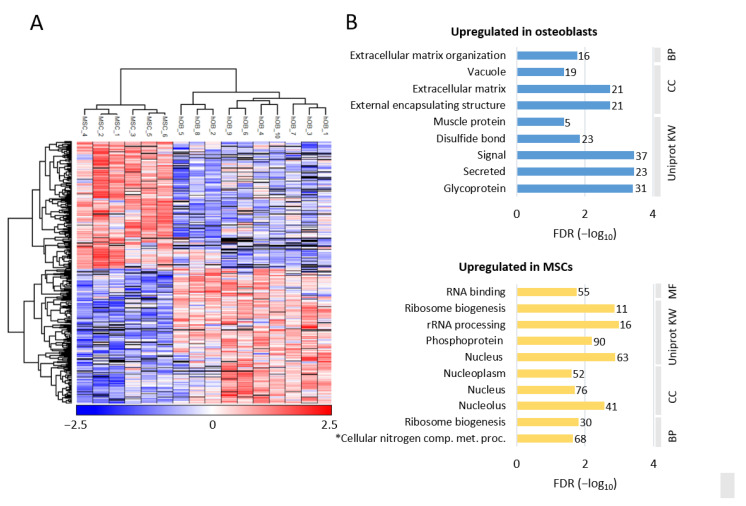
(page 7). Differential protein expression in osteoblasts and MSCs. (**A**) The figure shows the unsupervised hierarchical clustering analysis based on the 447 significantly abundant proteins when comparing cells from 10 osteoblast and six MSC donors. A total of 4747 proteins showed quantitative values (i.e., LFQ protein intensity) for at least three donors for each of the two cell types, and the unsupervised hierarchical clustering analysis was based on those 447 proteins that showed significant differences in fold change (FC) when comparing osteoblasts and MSCs; 231 of these proteins were increased in osteoblasts and 216 were increased in MSCs. The clustering analysis showed a clear separation between the groups, i.e., clearly separated the two cell types into two groups. The color key indicates the log_2_-transformed and Z-scored protein intensities. (**B**) Gene ontology enrichment of proteins with higher abundance in osteoblasts and MSCs. The analysis was performed in a GO tool. The bar plots show the the FDR (−log_10_) of enriched GO terms (i.e., biological processes, BP; cellular compartments, CC; molecular function, MF; and Uniprot Keyword, KW), and the number of proteins associated with each individual term is indicated to the left of the corresponding bar. * The full name of the term (lowest bar, Upregulated in MSCs) is Cellular nitrogen compound metabolic process.

We compared the proteomes for the ten osteoblast and the six MSC donors; this comparison was based on 4747 proteins that showed quantitative values (i.e., LFQ protein intensity) for at least three donors for each of the two cell types. The statistical analysis recognized 447 proteins that showed significant differences (i.e., *p* < 0.05) in fold change (FC); 231 of these proteins were increased in osteoblasts and 216 were increased in the MSCs (Supplementary file 2). Unsupervised hierarchical clustering analysis based on these 447 significantly differing proteins showed a clear separation of the two cell types (Figure 3A).

The results from GO enrichment analyses of the differentially abundant proteins are presented in Figure 3B. The proteins upregulated in osteoblasts were mainly annotated to extracellular matrix organization, external encapsulating structure, signal proteins, and glycoproteins. In contrast, the proteins upregulated in MSCs reflected increased levels of several proteins from various intracellular compartments, but especially nuclear and ribosomal proteins reflecting differences in transcription/translation, i.e., rRNA processing/ribosome biogenesis (Figure 3B).

Based on the QuickGO database, 60 proteins (64 including isoforms/protein groups) involved in differentiation/development of osteoblasts have been identified (child terms of GO:0001503, Ossification), and 11 of these proteins showed significant differences between osteoblasts and MSCs in our present study. Three additional proteins showing significant differences were identified based on the Uniprot database when using a similar strategy for protein selection (search word “osteoblast”). These 14 proteins are listed in Table S5 (corresponding gene names), together with the protein name and a brief description of the protein together with key words describing the most important/relevant biological functions. The majority of these proteins reflect osteoblastic differentiation or important functional characteristics of osteoblasts, including:

- Formation, modulation, or binding to extracellular matrix molecules: CYR61, ENPPI, FBN1, FHL2, GPNMB, ITGA11, and LOX.
- Bone mineralization: ENPP1, EPHA2, FBN1, FHL2, GPNMB, LOX, and TPM4.
- Interactions with integrins: CYR61, FHL2, and ITGA11.
- Intracellular signaling important for osteoblastic differentiation and/or functions: PTH signaling (CYR61, TMEM119), Wnt/β-catenin signaling (CYR61, FHL2, GTPBP4, ITGA11, TMEM119), BMP2 (CYR61, FBN1, TMEM119), and RhoA signaling (EPHA2, GTPBP4).
- Transcription: Only a minority of the proteins are regarded as transcriptional regulators (decreased levels of CEBPB, DDX21, EPHA2; increased levels of LOX), are important for cellular metabolism (FASN2) or are involved in exosomal communication between cells (EPHA2).

Taken together, these analyses based on differentially expressed proteins (Figure 3B) showed that osteoblasts and MSCs differed with regard to extracellular matrix, and our analysis of the osteoblast-associated proteins in addition shows that only a minority of the 447 differentially expressed proteins (i.e., 14 proteins) are known as osteoblast-specific or osteoblast-associated proteins.

**Figure 4:**
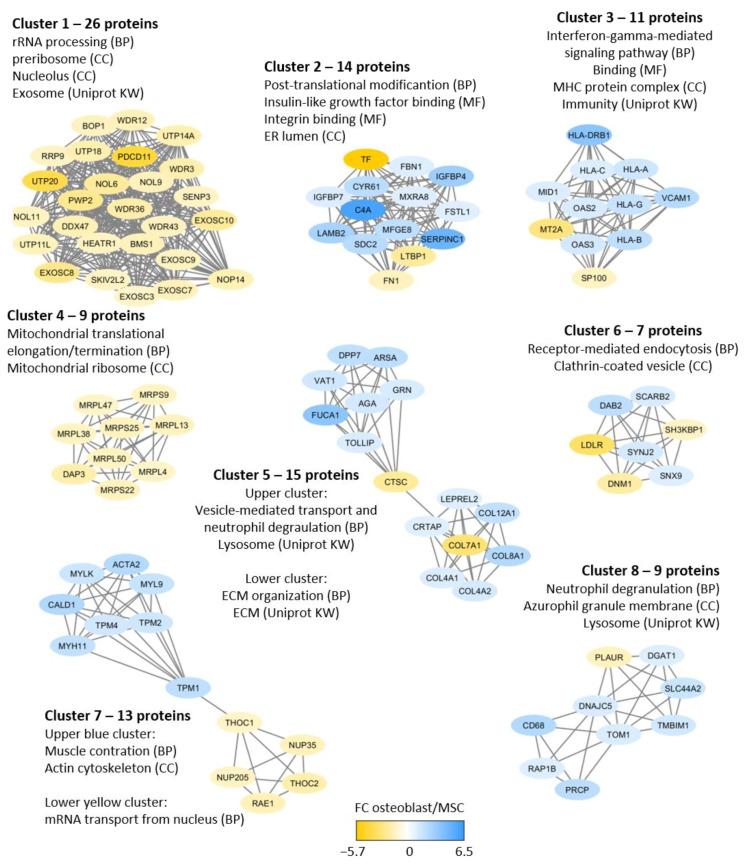
Protein interaction analysis based on 447 differentially regulated proteins. A total of 4747 proteins showed quantitative values (i.e., LFQ protein intensity) for at least three donors for each of the two cell types, and the interaction analysis was based on those 447 proteins that showed significant differences in fold change (FC) when comparing osteoblasts and MSCs; 231 of these proteins were increased in osteoblasts and 216 were increased in MSCs. The color key presents the protein fold change (FC) between osteoblasts and MSCs (based on normalized, log_2_-transformed and Z-scored protein intensities); increased protein levels in osteoblasts are marked with blue whereas increased levels in MSCs are marked with yellow. The figure presents the eight identified networks/clusters with high connectivity found by applying the MCODE application on the String network in Cytoscape. The GO/Uniprot Keyword terms (i.e., biological processes, BP; cellular compartments, CC; molecular function, MF; and Uniprot Keyword, KW) were obtained from String analyses.

The protein interaction analysis of the differentially expressed proteins (Figure 4) reflects the same cellular characteristics as the GO term analyses described above. We identified eight networks with high connectivity. Three of these networks included mainly proteins that were increased in MSCs and reflected differences in RNA processing/nuclear proteins (cluster 1, 26 proteins), mitochondrial functions (cluster 4, nine proteins), and RNA transport (lower part of cluster 7, a subset of five proteins). The five other networks mainly included proteins that showed increased expression in osteoblasts and thereby reflect biological characteristics of this cell type. These networks reflect high integrin/IGF signaling (cluster 2, 14 proteins), increased expression of HLA molecules (cluster 3, 11 proteins), differences in vesicle transport/degranulation/lysosome (cluster 5, 15 proteins), different cytoskeleton protein expression (upper part of cluster 7, 8 proteins) and different degranulation/lysosomes (cluster 8, 9 proteins). Finally, cluster 6 (endocytosis/clathrin, seven proteins) included similar numbers of proteins with increased abundance in both osteoblasts and MSCs. Thus, this network analysis reflects similar differences between osteoblasts and MSCs as described in Figure 3B, i.e., MSCs show increased levels of proteins involved in gene transcription/RNA processing together with cellular metabolism, whereas osteoblasts show high levels of proteins involved in endocytosis, intracellular transport, intracellular signaling, and cell surface molecules.

### 2.5. Only a Small Number of Osteoblast Proteins Show Significant Differences when Comparing Male Vs. Female Osteoblast Donors and Elderly Vs. Younger Donors

We compared the proteomic profiles for our male and female osteoblast donors (five donors in each group). Only 45 proteins differed significantly between these two donor groups (Table S6), and no significant GO-terms or protein interaction networks could be identified in analyses based on these 45 proteins (data not shown).

We also compared the proteomic profiles for osteoblasts derived from six donors above and four donors below 60 years of age. The median age for these two groups was 57 and 64 years, respectively. Only 39 proteins differed significantly between elderly and younger individuals (Table S7), and similar to the male/female comparison no significant GO-terms or protein interaction networks could be identified in analyses based on these 39 proteins (data not shown). We conclude that even though certain single proteins of potential interest could be identified both in the male/female (e.g., the osteoblast marker RUNX2) and the elderly/young comparison (e.g., the ligand/receptor pair GAS6/AXL), we could not detect any major differences in these comparisons of osteoblast proteomic profiles.

### 2.6. Osteoblast Responses to Extended Culture Including Suboptimal In Vitro Culture Conditions

Osteoblasts derived from six donors were further cultured in vitro, first in an optimized osteoblast medium and thereafter in ordinary serum-free Iscove’s Modified Dulbecco’s Medium (IMDM) medium for the last two days before cells were harvested (see Section 4.2). We compared the proteomic profiles for these cells after culture in IMDM medium with the original preculture osteoblasts. In our opinion, the ordinary serum-free IMDM should be regarded as a suboptimal medium compared with the optimized osteoblast cell culture medium, and for this reason we regard alteration of the proteomic profile of the IMDM cultured cells to reflect an in vitro stress response to a suboptimal microenvironment. A total of 3878 proteins had paired quantitative values for at least three osteoblast donors, and 286 of these proteins had significantly different abundance in the original optimal preculture and IMDM-cultured cells.

**Figure 5:**
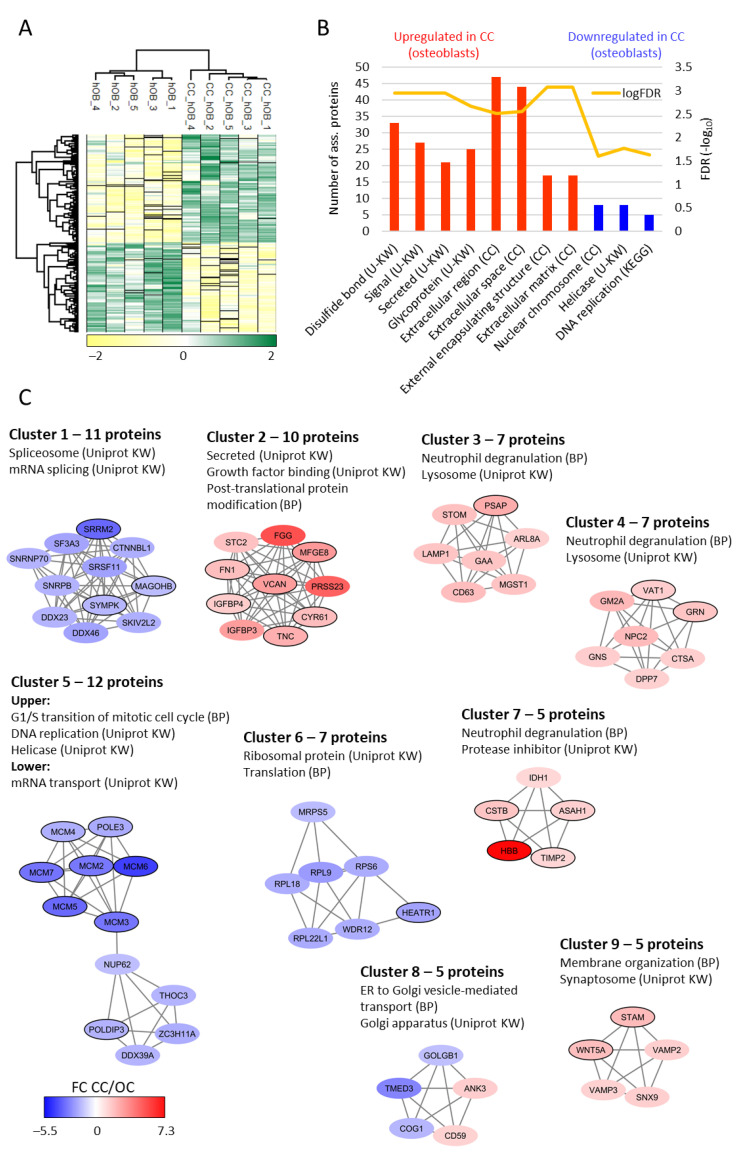
Comparison of osteoblast proteomic profiles before (i.e., original cells, OC) and after culture (referred to as cultured cells, CC) in suboptimal IMDM medium. A total of 286 proteins differed significantly between the two groups. (**A**) Hierarchical clustering based on the 286 proteins with significantly different abundance clearly separated the samples into two groups. The color key gradient indicates the normalized and log_2_-transformed LFQ protein intensity (Z-scored). (**B**) GO enrichment analysis of the 156 proteins with increased abundance after cell culture (red bars) and the 130 proteins with decreased abundance after cell culture (blue bars). The bar height represents the number of proteins associated to a given term (right *y*-axis) and the FDR (−log_10_) of each GO term is represented by the yellow line (left *y*-axis). (**C**) Protein–protein interaction network analysis was based on the 286 significantly altered proteins, generated in String and further processes with Cytoscape and MCODE to identify protein clusters with high connectivity. The color coding indicates the fold change (FC) after cell culture (CC) in suboptimal medium relative to original cells (OC), i.e., red protein nodes indicate increased abundance in suboptimal medium while blue protein nodes indicate decreased abundance in suboptimal medium. Proteins significantly regulated in both osteoblasts and MSCs are indicated by a black border surrounding the protein node (see Figure 6). Other abbreviations used in the figure: BP, biological process; CC, cellular compartment; U-KW, Uniprot key word; KEGG, Kyoto Encyclopedia of Genes and Genomes.

We did a hierarchical clustering analysis based on the 286 significantly different proteins, and this analysis clearly separated the original from the cultured cells (Figure 5A). GO enrichment analysis of the significantly up- and downregulated proteins showed that the suboptimal IMDM culture increased the abundance of proteins localized to the extracellular matrix and decreased the abundance of nuclear chromosome/helicase/DNA replication proteins (Figure 5B).

Protein–protein interaction network analysis supported the results from the GO enrichment analysis (Figure 5C). After suboptimal IMDM culture increased levels were observed for several proteins involved in extracellular release/binding (network 2, ten proteins), lysosomal proteins/degranulation (networks 3 and 4, each including seven proteins), protease regulation (network 7, five proteins) and intracellular trafficking (network 9, five proteins). In contrast, the proteins with significantly decreased levels are involved in splicing/mRNA metabolism (network 1, 11 proteins), DNA replication/helicase activity/mRNA transport (network 5, 12 proteins) and translation/ribosomal function (network 6, 7 protein). Finally, network 8 (five proteins) suggests that the Golgi apparatus/intracellular transport were also influenced by the culture conditions.

Taken together, the results from these analyses suggest that suboptimal IMDM culture influence important cellular functions with decreased levels, especially of proteins involved in transcription/translation and increased levels of proteins important for intracellular trafficking/organellar functions/extracellular release.

### 2.7. MSC Responses to Suboptimal In Vitro Culture Conditions

MSCs derived from five donors were also cultured in suboptimal IMDM medium (see Section 4.2), and we compared the proteomic profiles for MSCs before and after in vitro culture in IMDM. A total of 4192 proteins had paired quantitative values for at least three MSC donors, and 357 of these proteins had significantly different abundance in the original optimal preculture and suboptimal IMDM-cultured cells.

We first compared the IMDM-induced cell responses for osteoblasts and MSCs for 3677 proteins with quantitative values for at least three donors before and after cell culture in both datasets. A total of 87 proteins were significantly altered by suboptimal IMDM culture conditions in both cell types, and the Pearson correlation value for these common proteins was 0.94 (Figure S2).

**Figure 6:**
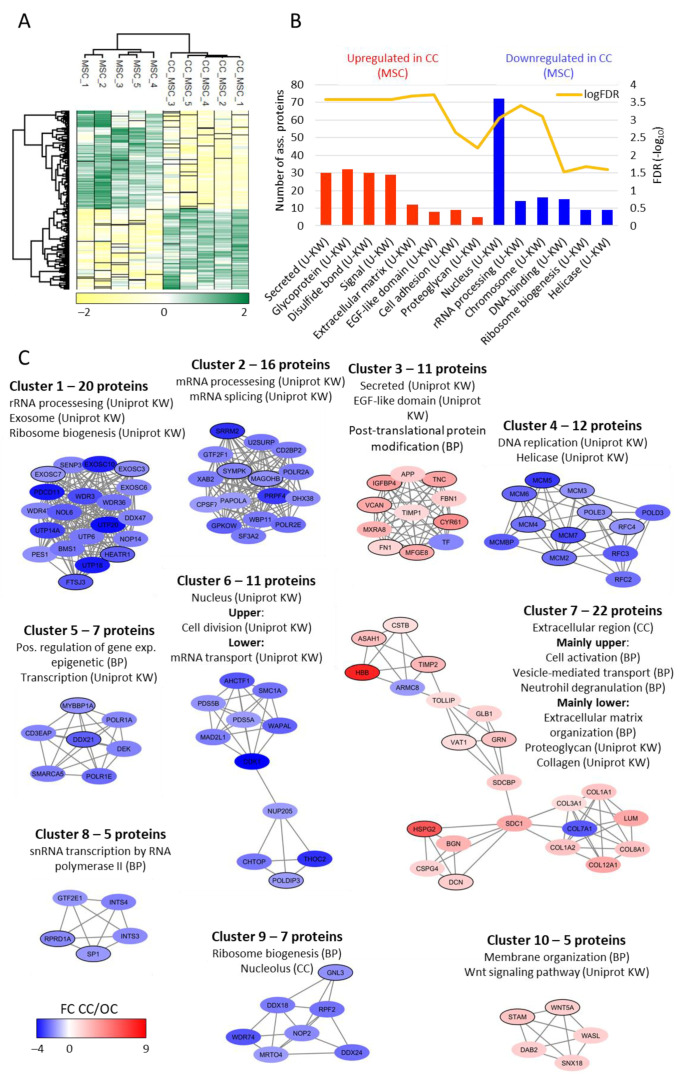
A comparison of MSCs proteomic profiles before and following in vitro culture in suboptimal serum-free IMDM medium; the suboptimal in vitro microenvironment causing increased levels especially of several proteins involved in extracellular matrix, cell adhesion, and EGF-like domain after cell culture in suboptimal medium. A total of 4192 proteins had paired quantitative values for at least three MSC donors, and 357 of these proteins had significantly different abundance when comparing original optimal preculture (i.e., original cells, OC) and suboptimal IMDM-cultured cells (i.e., cultured cells, CC). (**A**) Unsupervised hierarchical clustering analysis based on the 357 proteins with significantly different abundance when comparing MSCs cells before and after cell clearly separated the samples into two groups. The color key gradient indicates the normalized and log_2_-transformed LFQ protein intensity (Z-scored). (**B**) GO enrichment analysis of the 162 proteins with increased abundance after cell culture (red bars) and the 195 proteins with decreased abundance after cell culture (blue bars). The Uniprot Keyword (KW) terms are presented. The bar height represents the number of proteins associated to a given term (right *y*-axis) and the FDR (−log_10_) of each GO term is represented by the yellow line (left *y*-axis). (**C**) Protein–protein interaction network analysis was based on the 357 significantly altered proteins, generated in String and further processes with Cytoscape and MCODE to find protein clusters with high connectivity. The color coding indicates the fold change (FC) after cell culture (CC) in suboptimal medium relative to original cells (OC); i.e., red protein nodes indicate increased abundance in suboptimal medium while blue protein nodes indicate decreased abundance in suboptimal medium. Proteins significantly regulated in both osteoblasts and MSCs (see Figure 5) are indicated by a black border surrounding the protein node. Abbreviations used in the figure: BP, biological process; CC, cellular compartment; Uniprot KW, Uniprot keyword.

Hierarchical clustering, GO enrichment, and protein–protein interaction analyses of the 357 MSC proteins that were significantly altered by a suboptimal in vitro microenvironment showed several similarities with the osteoblasts (Figure 6A–C); proteins involved in intracellular trafficking/organellar functions were increased and proteins involved in transcription/translation were decreased after culture in IMDM. However, the GO enrichment analysis indicated that nuclear proteins are more affected (i.e., downregulated) in MSCs than osteoblasts (Figure 6B), whereas proteins annotated to the EGF-like domain are upregulated. Furthermore, protein interaction network analysis based on the 357 MSC proteins showed downregulation of nuclear proteins involved in cell division (cluster 6, 11 proteins) and ribosome biogenesis (cluster 9, seven proteins) (Figure 6C). Upregulation of collagens also seem to be specific for the MSCs (cluster 7, lower part).

## 3. Discussion

Osteoblasts and MSCs are important bone marrow stromal cells [1,2]. Both cell types are important for the formation of the stem cell niches, although they differ in their stem cell supporting functions [2]. In this context we did a proteomic comparison of normal osteoblasts and MSCs derived from healthy individuals.

Our osteoblasts and MSCs were prepared by standardized separation procedures and in vitro culture before cryopreservation, and fundamental cellular characteristics were documented (distributor’s information). During further in vitro culture the cells proliferated, the large majority of cells were viable until the end of the culture period, and more than 4000 proteins could be quantified for all cell populations. Our proteomic studies identified many osteoblast-specific or osteoblast-associated proteins, and the levels for several of them are known to correlate with the levels of conventional osteoblastic marker, e.g., alkaline phosphatase (see Tables S2, S3, and S5). However, it should be emphasized that although our osteoblast-specific proteins (Tables S2 and S3) could not be quantified in any of our MSCs populations, these proteins may also be present at lower concentrations in MSCs and therefore may have been quantified if sample fractionation or more sensitive mass spectrometry instrumentation had been used. Taken together, these observations further document the purity of our cell populations.

Most proteins did not differ significantly when comparing osteoblasts and MSCs, and this is not surprising because osteoblasts differentiate from MSCs [9,10,11]. MSCs showed increased levels of several transcriptional regulators (Table S4); this is similar to normal and leukemic hematopoietic stem cells that also show increased expression of several transcriptional regulators compared with their more mature cells [23].

Osteoblasts are important for bone formation/remodeling and they support proliferation and survival of both normal [5,6,7,8] and malignant cells in the bone marrow [12,13,14,15,16,17,18,24,25,26]. Therapeutic targeting of osteoblasts is thus considered for several diseases. To reduce the risk of side effects it will then be important to target molecular mechanisms that are specific or particularly important for osteoblasts. Our present characterization and comparison of proteomic profiles in osteoblasts and MSCs may contribute to a scientific basis for selection of more osteoblast-specific therapeutic targets.

We identified several osteoblast-specific proteins that are important for osteoblastic differentiation and/or functions, including extracellular matrix proteins, regulators of ossification/mineralization, mediators involved in Wnt, Notch, and integrin signaling, and an osteoblast-specific interaction network. Our results presented in Table 1 and Table S2 and Figure 3 show that the following osteoblast-specific/associated proteins were identified:

- *Osteoblast differentiation:* BST2, CHI3L1, FBN2, FYN, PRKCQ, PRUNE2, PSEN2, VEGFC.
- *Transcriptional regulation:* Regulators or targets of Runx2 (EPHB4, SPP1) and NFκB, (PRUNE2, PSEN2). LMAA is also regarded as a transcriptional regulator.
- *Intracellular signaling pathways:* the network included regulators of Wnt (BRI3), BMP (BST2, FBN2, CHMP1B, MAP3K7), RhoA (EPHB4, PRUNE2), Notch (PRUNE2, PSEN2), TGF (FBN2, ELN, MAP3K7) and TNF signaling (MAP3K7, TANK, TRAF6).
- *Intracellular trafficking/transport.* Osteoblasts are characterized by an extensive Golgi apparatus and endoplasmatic reticulum [27], and the interaction network included proteins that are important for the Golgi system (B4GALT5, FAM20A), intracellular trafficking (CHMP2B), and cytoskeleton (KRT77, KRT80).
- *Bone metabolism/mineralization:* B4GALT5, ELN, FBN2, TLR4, EPHB4, FGG.
- *Bone remodeling/homeostasis:* EPHB4, LMNA, TRAF6, TANK, SPP1, VEGFC.

Thus, these well-characterized osteoblastic proteins [20,21,22,28,29,30]) should not only be regarded as single molecules but as parts of a complex osteoblast-specific protein interaction network.

Immunoregulatory mechanisms are involved in the regulation of bone formation and remodeling [31,32,33,34]. First, the crosstalk with the immune system is illustrated by the process of fracture healing where γδ and CD8^+^ T cells together with B cells seem to be involved [31]. Second, several proinflammatory cytokines are involved in regulation of osteoblast differentiation and the balance between osteoblasts and osteoclasts [32,33,35], and these mediators seem to be important also for disease-associated osteoporosis [32,33]. Third, TLR4 initiated signaling is involved in the regulation of osteoblast differentiation and function [32,34]. Several such immunoregulatory mediators are also included in an osteoblast-specific protein–protein interaction network (see Figure 2; CD14, TANK, TRAF6) together with immunoregulatory HLA molecules [36]. Thus, our osteoblast-specific protein interaction network seems to be involved in a crosstalk between osteoblasts and regulators of inflammation/immunity.

Our protein network analyses (Figure 4) confirmed the differences between osteoblasts and MSCs suggested by the corresponding GO term analyses. Several network proteins reflect osteoblastic differentiation and functions, e.g., high osteoblast levels of proteins involved in IGF1 and integrin-induced signaling, immunoregulation (e.g., HLA molecules) and endocytosis. Furthermore, the osteoblast-specific/associated proteins identified in our present study included several extracellular matrix molecules, and similar differences are also reflected in the osteoblast/MSCs secretomas [37]. Several osteoblast-associated networks involving the actin cytoskeleton, vesicle-mediated transport, degranulation, and extracellular matrix organization (see Figure 4) may also be important for extracellular matrix protein release by osteoblasts.

Pharmacological targeting of osteoblasts or the balance between osteoblasts and osteoclasts is already an accepted strategy for the treatment of osteoporosis, and the FDA-approved osteoporosis drugs target RANKL, parathyroid hormone, and sclerostin [20]. Other possible therapeutic targets in osteoporosis are αVβ3 integrin [38], the cathepsin K protease [39] and the Wnt-inactivating Notum [40]. Pappalysin 1 was also identified as an osteoblast-specific protein in our present study (Table S1), and mutations in this gene are associated with a skeletal phenotype (i.e., short stature) suggesting that this protein is essential for normal osteoblastic function(s) [41]. Our present study characterizes the biological/molecular context for several of these (possible) therapeutic targets, including PTH analogues and Wnt signaling.

Previous studies have described age- and sex-dependent differences both for osteoblasts and MSCs [37,42]. This could not be observed in our present study even though we have previously described such secretomal variations [37], but a previous study also showed that differences in constitutive extracellular protein release are not necessarily reflected in cellular proteomic profiles [43]. Other possible explanations could be that our present study included relatively few donors and/or the relatively small variation in donor age for our osteoblasts (median age 62.5 years, range 54–73 years). Finally, our MSC donors also had a similar variation (median age 62.5 years, range 47–70 years), and our results should therefore be interpreted with care and may be representative only for this age group.

We compared the proteomic profiles of the original cryopreserved osteoblasts and MSCs with cells derived after extended in vitro culture including serum-free medium IMDM without additional supplementation; we regard this IMDM medium as suboptimal for osteoblast/MSC culture. The IMDM medium was chosen because it has previously been used for culture of cell populations including stem cells, and comparable media have also been used for culture of cells with osteoblastic phenotypes [44,45,46,47]. Our results show that this extended culture period including suboptimal culture conditions alters the proteomic profiles of both osteoblasts and MSCs compared with the original cryopreserved cells. In our opinion these culture-induced alterations should be regarded as specific for the in vitro conditions used in our present study, and the effect of other culture conditions will probably differ. However, our results clearly illustrate the importance of using highly standardized methodological procedures when osteoblasts/MSCs are tried in clinical practice, and a careful characterization of the cells (possibly including proteomic profiling) will then be necessary for cells used in patient treatment.

Both osteoblasts and MSCs have been tried in the treatment of human disease, including cell therapy and the use of conditioned medium [48,49,50]. Conditioned medium derived from MSC-differentiated osteoblasts seems to improve engraftment of cord blood progenitors [51], stem cell derived exosomes can improve osteoporosis by promoting osteoblast proliferation [52], and injection of in vitro expanded osteoblasts has been tried in the treatment of fractures [9,53], as well as osteonecrosis [54]. Furthermore, animal models suggest that MSC transplantation is effective in the treatment of osteoporosis [55], stem cell-derived osteoblast-like cells can prevent glucocorticoid-induced bone loss [56], and ex vivo manipulated osteoblast-like cells can support bone regeneration [57]. Finally, MSC/osteoblast transplantation has also been tried as a part of allogeneic stem cell transplantation [58], and various cell-free alternatives based on ex vivo manipulation of MSCs are also considered for muscle trauma and regenerative medicine [59,60,61]. Taken together, these studies suggest that osteoblast therapy should be further investigated as a possible strategy in the treatment of bone and bone marrow diseases.

## 4. Materials and Methods

### 4.1. Human MSCs and Osteoblasts

Cryopreserved human MSCs (at least 500,000 cells) derived from the bone marrow of 10 healthy donors and human osteoblasts (at least 500,000 cells) derived from the hip femoral tissue of 6 healthy donors were purchased from PromoCell GmbH (Heidelberg, Germany; reference codes C-12974 and 12720, respectively) (Table 3). The 10 osteoblast donors included five men and five women (median age 62.5 years, range 53–73 years), whereas the six MSC donors included four men and two women (median age 62.5 years, range 47–70 years). The cells had been cryopreserved in passage two after being characterized by flow cytometric analysis and in addition examined for morphology, proliferative capacity, adherence rate and viability.

According to the manufacturer, MSCs were isolated from the bone marrow of femoral heads and plated in MSC growth medium 2 (Promocell, reference code C-28009) (with 2% serum) and attached within 1–2 days. The osteoblasts were isolated using explant technique (i.e., spongy bone were cut into small pieces, washed and treated with collagenase to remove potential fibroblasts) before being plated with Osteoblast growth medium (Promocell, reference code C-27001) (with 10% serum). Osteoblasts will then grow out of the bone, attach to plastic and proliferate after 1–2 weeks. When subconfluent, they are detached and grown in a secondary culture. Furthermore, both cell types were washed, trypsinized, and finally exposed to trypsin-neutralizing solution (Promocell, reference code C-41100) (0.05% trypsin inhibitor in 0.1% BSA) and centrifuged before cryopreservation at passage 2 using Cryo-SFM media (Promocell, reference code C-29910), which is a protein-free and animal-component-free media based on methylcellulose, DMSO and other cryoprotectants. All samples showed a similar high viability and proliferative capacity before freezing and were cryopreserved according to the same standardized procedure. All the MSCs were in addition tested for their ability to differentiate into mesenchymal lineages, and all osteoblast samples were tested for expression of alkaline phosphatase and capacity of mineralization (manufacturer’s information). Mycoplasma infections could not be detected for any sample. The samples were stored in liquid nitrogen and later handled according to the manufacturer’s instructions.

### 4.2. Preparation of Cell Samples: Original Cells and In Vitro Incubated Cell Samples

MSC and osteoblast samples were thawed at 37 °C by using a highly standardized procedure recommended by the manufacturer. At least 350,000 cells were thereafter centrifuged before the cell pellet was lysed in 130 µL lysis buffer (4% SDS/0.1 M Tris-HCl, pH 7.6) as described previously [62]. These cells will be referred to as original cells.

For five osteoblast and five MSC samples at least 150,000 cells were further incubated (37 °C, 5% CO_2_, humidified atmosphere) in T25 flasks (Falcon, Glendale, AZ, USA) with Mesenchymal Stem Cell Growth Medium (PromoCell, reference code C-28009) or Osteoblast growth medium (PromoCell, reference code C-27001) with supplementMix (PromoCell, reference code C-39615). The medium was exchanged after 24 h. When cells had reached 70% confluence, the cell were washed twice in 5 mL 1× PBS before the medium was exchanged with serum-free, no phenol red IMDM medium (Gibco, New York, NY, USA; reference code 21056023); the 70% confluence was reached after 4–5 days except for the cells derived from hOB donor 2 (cultured for 6 days) and MSC donor 4 (cultured for 13 days). The cells were incubated for additional 48 h in the serum-free medium before they were lysed in 1 mL lysis buffer. All cultures were controlled by light microscopy during and at the end of incubation, and the dominant cell morphology for all cultures at all time points were viable, adherent bright cells.

### 4.3. Proteomics Sample Preparation

The single-pot, solid-phase-enhanced sample preparation (SP3) [63] method was used for protein digestion of 5–10 µg SDS-lysed samples, with the addition of one extra cleaning with SP3 beads for SDS removal. As a verification of the sample quality, the NanoDrop UV-Vis spectrophotometer (Thermo Scientific, Waltham, MA, USA) was used to measure peptide concentration prior to LC-MS/MS analysis.

### 4.4. Liquid Chromatography (LC) Tandem Mass Spectrometry (MS) Analysis

For each sample, 0.5 µg tryptic peptides dissolved in 2% acetonitrile (ACN) and 0.5% formic acid (FA) were injected into an Ultimate 3000 Rapid Separation LC system (Thermo Scientific) coupled to a QExactive HF mass spectrometer (MS) (Thermo Scientific, Bremen, Germany) using settings as described in detail previously [43], except the LC gradient composition was shorter, i.e., 5% solvent B (i.e., 100% ACN) during trapping over 5 min followed by 5–8% B for 0.5 min, 8–24% B for the next 65.5 min, 24–35% B for 24 min, and 35–90% B for 5 min, hold 90% B for 8 min and ramp to 5% B for 5 min, before keeping 5% B for 20 min.

### 4.5. Statistical and Bioinformatical Analyses

The LC-MS raw files were searched in MaxQuant (version 1.6.1.0, Max Planck Institute for Biochemistry, Martinsread, Germany) [64] using the same settings as in [37], except alignment window was set to 0.7 and ITMS/MS to 0.5. Perseus (version 1.6.1.1, Max Planck Institute for Biochemistry) was used to process and filter the MaxQuant results [65]. Proteins with at least three valid values (LFQ protein intensity value) in each group were selected for statistical comparisons using Welch’s *t*-test and *Z*-statistics [66]. Proteins with at least three cultured cells/original cells fold change pairs were selected for analyses by paired *t*-test and *Z*-statistics. Proteins with *p*-values < 0.05 based on both the *t*-test and Z-statistics were considered as being significantly different abundant.

Relevant/important GO terms were retrieved from QuickGO (https://www.ebi.ac.uk/QuickGO/, accessed on 30 March 2021, EMBL-EMI, Cambridge, United Kingdom) and the gene names were compared to the present data. Hierarchical clustering was performed in Perseus with Pearson correlations as distance metrics and complete linkage. GO enrichment analyses were performed using a GO tool [67] (accessed on 26 April 2021, Department of Systems biology/ Metagenomics, Institute for Molecular Life Sciences, University of Zurich, Zürich, Switzerland) with the GO slim subset. The enrichment method “characterize foreground” was used for the global proteome analysis (Section 2.1.) and “compare samples” for all other analyses. GO terms with FDR < 0.05 were considered significantly enriched. Protein–protein interaction networks were generated by the String database (version 11.0, STRING Consortium 2020) [68] and processed in Cytoscape (version 3.3.0., the Cytoscape Consortium, San Diego, CA, USA) [69] with the MCODE application (version 1.4.2. Bader lab, Toronto, Canada) [70] as described previously [71].

## 5. Conclusions

Osteoblasts can differentiate from MSCs, and pharmacological targeting or transplantation of MSCs/osteoblasts are now considered for the treatment of uman diseases. Our comparison of the proteomic profile showed a large overlap between the two cell types, but we also identified an osteoblast-specific protein–protein interaction network that influences and integrates both inflammatory signaling and regulatory mechanisms involved in the differentiation and function of osteoblasts. Furthermore, the osteoblast and MSC proteomic profiles are modulated by in vitro culture conditions, and pretreatment ex vivo manipulation of both osteoblasts and MSCs will therefore require careful characterization and standardization of culture conditions.

## Figures and Tables

**Table 1:** Proteins quantified only in osteoblasts and reflecting the regulation of their differentiation and/or specialized functions. The table presents the osteoblast-specific proteins (i.e., corresponding gene names) included in the selected GO terms obtained from QuickGO. The numbers given in paretheses (left column) represent the total number of proteins quantified in our present study and included in the GO term together with the total number of gene products from the UniprotKB database belonging to each of the GO terms. Duplicates (i.e., protein isoforms) are removed from both the QuickGO list and the present dataset.

GO Term	Name	Matching Protein in Osteoblasts Only
GO:0031012 (198/700)	Extracellular matrix (CC)	21 proteins: ANGPTL3, APOA1, APOE, BCAM, CHI3L1, COL5A3, CRISPLD2, CXCL12, DMBT1, ELN, F9, FBN2, FGG, LGALS9, LRRC32, MFAP2, MUC5B, NTN4, PODN, TFPI2, TINAGL1
GO:0030198 (159/404)	Extracellular matrix organization (BP)	8 proteins: COL5A3, CRISPLD2, ELN, FBN2, FGG, MFAP2, NTN4, SPP1
GO:0001503 (105/297)	Ossification (BP)	SPP1, TRAF6
GO:0030278 (29/122)	Regulation of ossification	FBN2
GO:0045667 (39/127)	Regulation of osteoblast differentiation	FBN2, HDAC7, JAG1, VEGFC
GO:0007229 (48/203)	Integrin-mediated signaling pathway	ANGPTL3, APOA1
GO:2001044 (10/15)	Regulation of integrin-mediated signaling pathway	LIMS2
GO:0016055 (140/442)	Wnt signaling pathway	DAAM2, MAP3K7
GO:0030111 (144/406)	Regulation of Wnt signaling pathway	APOE, DAAM2, IGFBP2
GO:0007219 (34/165)	Notch signaling pathway	JAG1, PSEN2
GO:0008593 (27/110)	Regulation of Notch signaling pathway	JAG1

**Table 2:** Classification of osteoblast-specific proteins (i.e., proteins reaching quantifiable levels only for osteoblasts but not for any MSC donor) using a GO tool. The Uniprot keywords reaching statistical significance are listed together with the corresponding s-value, foreground count and False Discovery Rate (FDR). S-value is a combination of (minus log) *p* value and effect size (i.e., positive associations in the foreground divided by all associations); a positive value indicates overrepresentation of a given term, and a negative value indicates underrepresentation of a given term. Foreground counts indicate the number of positive associations for a given term (i.e., the number of proteins associated with the given term).

Uniprot Keyword	S-Value	Foreground Count	FDR
Signal	0.69	25	0.0008
Disulfide bond	0.66	24	0.0008
Nucleus	−0.57	6	0.003
Glycoprotein	0.55	25	0.002
Phosphoprotein	−0.45	24	0.040
Acetylation	−0.45	13	0.022
Secreted	0.39	16	0.002
Cell membrane	0.24	18	0.047
Adaptive immunity	0.17	6	0.003

**Table 3:** The characteristics of the MSC and osteoblast donors. Donors marked with an * asterisk were not included in the cell culture experiments. All samples were obtained from Caucasian donors. Abbreviations: MSC, mesenchymal stem cells; hOB, human osteoblasts.

Donors	Gender	Age (Years)	Lot Number
**Osteoblasts**			
hOB Donor 1	Male	54	422Z050
hOB Donor 2	Male	58	422Z047.2
hOB Donor 3	Male	63	415Z007.2
hOB Donor 4	Female	71	443Z004.2
hOB Donor 5	Female	62	413Z026.2
* hOB Donor 6	Female	73	427Z010.2
* hOB Donor 7	Male	58	422Z051
* hOB Donor 8	Male	64	427Z036
* hOB Donor 9	Female	64	422Z031.2
* hOB Donor 10	Female	56	415Z011
**MSCs**			
MSC Donor 1	Male	47	402Z027
MSC Donor 2	Male	62	413Z021.4
MSC Donor 3	Male	63	411Z011.4
MSC Donor 4	Female	57	409Z018.1
MSC Donor 5	Female	66	421Z029.3
* MSC Donor 6	Male	70	429Z022

## Data Availability

The data presented in this study are available on request from the corresponding author.

## Supplementary Materials

The following are available online at https://www.mdpi.com/article/10.3390/ijms22115665/s1, Supplementary file 1: Figure S1: Network analysis of MSC-specific proteins, i.e., proteins only quantified for MSCs but not for any of the osteoblast population, Figure S2: Scatter plot of the fold change (FC) for cells cultured in suboptimal serum-free IMDM medium (CC, cultured cells) vs. the original cell (OC); a presentation of the fold changes (FCs) for osteoblasts and MSCs, Table S1: Proteins expressed only in osteoblasts, Table S2: Proteins involved in differentiation and development of osteoblasts, Table S3: Description of proteins expressed only in osteoblasts and included in the osteoblast-specific protein interaction network, Table S4: Proteins expressed only in MSCs, Table S5: Proteins involved in differentiation/development of osteoblasts and showing significant differences when comparing osteoblasts with MSCs, Table S6: Proteins showing a significant difference between male and female osteoblast donors, Table S7: Proteins showing a significant difference between osteoblast donors above and below 60 years of age. Supplementary file 2: Quantitative raw data from MaxQuant, processed main data, list of proteins with significantly different abundance in MSC versus osteoblasts.
