# Supplementary material for: Proteomic Comparison of Bone Marrow Derived Osteoblasts and Mesenchymal Stem Cells
Source: Int J Mol Sci. 2021 May 26;22(11):5665. doi: 10.3390/ijms22115665 (PMC8198503; doi:10.3390/ijms22115665)
Supplement: Supplementary file 1 [file ijms-22-05665-s001.zip › ijms-1227618-suppl-english.pdf]

Supplementary Material

Table S1. Proteins expressed only in osteoblasts. The table gives a list (corresponding gene name, protein name) of all proteins that could be quantified in osteoblasts but were not detected for any of the MSC donors.

Gene Name	Protein Name
ACTC1	Actin, alpha cardiac muscle 1
ADA	Adenosine deaminase
SCIN	Adseverin
AIF1	Allograft inflammatory factor 1
ANGPTL3	Angiopoietin-related protein 3
ACE	Angiotensin-converting enzyme;Angiotensin-converting enzyme, soluble form
APOA1	Apolipoprotein A-I;Proapolipoprotein A-I;Truncated apolipoprotein A-I
APOE	Apolipoprotein E
ATXN3	Ataxin-3
NPR3	Atrial natriuretic peptide receptor 3
AURKA	Aurora kinase A
BCAM	Basal cell adhesion molecule
BCL7C	B-cell CLL/lymphoma 7 protein family member C
B3GALTL	Beta-1,3-glucosyltransferase
B4GALT5	Beta-1,4-galactosyltransferase 5
GUSB	Beta-glucuronidase
LACTB2	Beta-lactamase-like protein 2
BST2	Bone marrow stromal antigen 2
BRI3	Brain protein I3
BRD4	Bromodomain-containing protein 4
KCTD3	BTB/POZ domain-containing protein KCTD3
C4BPA	C4b-binding protein alpha chain
CAMSAP1	Calmodulin-regulated spectrin-associated protein 1
NTPCR	Cancer-related nucleoside-triphosphatase
CADM1	Cell adhesion molecule 1
CDCA2	Cell division cycle-associated protein 2
PDE5A	cGMP-specific 3,5-cyclic phosphodiesterase
CHMP1B	Charged multivesicular body protein 1b
CHMP2B	Charged multivesicular body protein 2b
CHI3L1	Chitinase-3-like protein 1
F9	Coagulation factor IX
COL5A3	Collagen alpha-3(V) chain
CRISPLD2	Cysteine-rich secretory protein LCCL domain-containing 2
DMBT1	Deleted in malignant brain tumors 1 protein
DAAM2	Disheveled-associated activator of morphogenesis 2
HECTD3	E3 ubiquitin-protein ligase HECTD3
RNF13	E3 ubiquitin-protein ligase RNF13
ENTPD1	Ectonucleoside triphosphate diphosphohydrolase 1
ENPP2	Ectonucleotide pyrophosphatase/phosphodiesterase family member 2
EFHD1	EF-hand domain-containing protein D1
ELN	Elastin
EPHA4;EPHA5;EPHA3	Ephrin type-A receptor 4;Ephrin type-A receptor 5;Ephrin type-A receptor 3
EPHB4;EPHB3	Ephrin type-B receptor 4;Ephrin type-B receptor 3
FBN2	Fibrillin-2
FGG	Fibrinogen gamma chain
FBP2	Fructose-1,6-bisphosphatase isozyme 2
FUNDC2	FUN14 domain-containing protein 2

GALT	Galactose-1-phosphate uridylyltransferase
LGALS9	Galectin-9
HEATR5A	HEAT repeat-containing protein 5A
HCLS1;PCMTD2;OTUD6A	Hematopoietic lineage cell-specific protein
HGFAC	Hepatocyte growth factor activator
HMG20A	High mobility group protein 20A
HNMT	Histamine N-methyltransferase
HDAC7	Histone deacetylase 7
HLA-A	HLA class I histocompatibility antigen, A-23 alpha chain
HLA-A	HLA class I histocompatibility antigen, A-30 alpha chain
HLA-A	HLA class I histocompatibility antigen, A-36 alpha chain
HLA-A	HLA class I histocompatibility antigen, A-68 alpha chain
HLA-F	HLA class I histocompatibility antigen, alpha chain F
HLA-B	HLA class I histocompatibility antigen, B-45 alpha chain
HLA-C	HLA class I histocompatibility antigen, Cw-1 alpha chain
HLA-C	HLA class I histocompatibility antigen, Cw-18 alpha chain
HLA-DRB4	HLA class II histocompatibility antigen, DR beta 4 chain
HLA-DRB1	HLA class II histocompatibility antigen, DRB1-11 beta chain
HLA-DRB1	HLA class II histocompatibility antigen, DRB1-15 beta chain
HLA-DRB1;HLA-DRB5	HLA class II histocompatibility antigen, DRB1-16 beta chain
IGHA1	Ig alpha-1 chain C region
IGKC	Ig kappa chain C region
IGFBP2	Insulin-like growth factor-binding protein 2
IFT140	Intraflagellar transport protein 140 homolog
KRT77	Keratin, type II cytoskeletal 1b
KRT80	Keratin, type II cytoskeletal 80
KYNU	Kynureninase
LPHN2	Latrophilin-2
LMOD1	Leiomodin-1
LRRC32	Leucine-rich repeat-containing protein 32
LRRC58	Leucine-rich repeat-containing protein 58
LIMS2	LIM and senescent cell antigen-like-containing domain protein 2
LCN1	Lipocalin-1
LYZ	Lysozyme C
MROH1	Maestro heat-like repeat-containing protein family member 1
NIPA1	Magnesium transporter NIPA1
MEST	Mesoderm-specific transcript homolog protein
STEAP4	Metalloreductase STEAP4
MFAP2	Microfibrillar-associated protein 2
MAP3K7	Mitogen-activated protein kinase kinase kinase 7
SLC16A5	Monocarboxylate transporter 6
CD14	Monocyte differentiation antigen CD14
MUC5B	Mucin-5B
NADK	NAD kinase
NTN4	Netrin-4
SPP1	Osteopontin
PALMD	Palmdelphin
PAPPA	Pappalysin-1
PALM	Paralemmin-1
SPG7	Paraplegin
PDDC1	Parkinson disease 7 domain-containing protein 1
PBLD	Phenazine biosynthesis-like domain-containing protein
FXYD1	Phospholemman

PHYHD1	Phytanoyl-CoA dioxygenase domain-containing protein 1
PEAR1	Platelet endothelial aggregation receptor 1
CD36	Platelet glycoprotein 4
PODN	Podocan
PIGR	Polymeric immunoglobulin receptor;Secretory component
LMNA	Prelamin-A/C;Lamin-A/C
PSEN2	Presenilin-2;Presenilin-2 NTF subunit;Presenilin-2 CTF subunit
ATP9A	Probable phospholipid-transporting ATPase IIA
BICD1	Protein bicaudal D homolog 1
C12orf4	Protein C12orf4
FAM20A	Protein FAM20A
JAG1	Protein jagged-1
PRKCQ	Protein kinase C theta type
PRUNE2	Protein prune homolog 2
SAMD4A	Protein Smaug homolog 1
UNC13B	Protein unc-13 homolog B
TGM3	Protein-glutamine gamma-glutamyltransferase E
SLC46A1	Proton-coupled folate transporter
PIPSL	Putative PIP5K1A and PSMD4-like protein
DHX32	Putative pre-mRNA-splicing factor ATP-dependent RNA helicase DHX32
MRAS	Ras-related protein M-Ras
RAB27B	Ras-related protein Rab-27B
RGN	Regucalcin
RTN4	Reticulon-4
ARHGAP24	Rho GTPase-activating protein 24
RNF121	RING finger protein 121
SSPN	Sarcospan
CD163L1	Scavenger receptor cysteine-rich type 1 protein M160
DCLK1	Serine/threonine-protein kinase DCLK1
SLC17A5	Sialin
SIPA1L3	Signal-induced proliferation-associated 1-like protein 3
SLIT3	Slit homolog 3 protein
SNX21	Sorting nexin-21
STAB1	Stabilin-1
CXCL12	Stromal cell-derived factor 1;SDF-1-beta(3-72);SDF-1-alpha(3-67)
SYNPO2	Synaptopodin-2
TAPBPL	Tapasin-related protein
TBXAS1	Thromboxane-A synthase
TFPI2	Tissue factor pathway inhibitor 2
TRAF6	TNF receptor-associated factor 6
TANK	TRAF family member-associated NF-kappa-B activator
EBF3	Transcription factor COE3
TM4SF1	Transmembrane 4 L6 family member 1
TRIM47	Tripartite motif-containing protein 47
TINAGL1	Tubulointerstitial nephritis antigen-like
TNFAIP2	Tumor necrosis factor alpha-induced protein 2
TNFAIP8	Tumor necrosis factor alpha-induced protein 8
TNFRSF12A	Tumor necrosis factor receptor superfamily member 12A
TPD52L1	Tumor protein D53
FYN	Tyrosine-protein kinase Fyn
UPP1	Uridine phosphorylase 1
VEGFC	Vascular endothelial growth factor C
KCNAB2	Voltage-gated potassium channel subunit beta-2

WDR81	WD repeat-containing protein 81
WISP2	WNT1-inducible-signaling pathway protein 2
ZNF281	Zinc finger protein 281

Table S2. Proteins involved in differentiation and development of osteoblasts. The table shows a list of proteins that reached detectable levels only in osteoblasts, included in the GO terms GO:0045667 Regulation of osteoblastic differentiation, GO:22001044 Integrin-mediated signaling pathway, GO:0030509 BMP signaling pathway, GO:0030510 Regulation of BMP signaling pathway, GO:0031012 Extracellular matrix (Cellular compartment), GO:0030198 Extracellular matrix organization (biological process), GO:0001503 Ossification, GO:0030278 Regulation of ossification, GO:0016055 Wnt signaling pathway and GO0030111 Regulation of Wnt signaling pathway. The table shows the corresponding gene name and the protein name together with a brief description and keywords reflecting important characteristics of the proteins. The information is based on Uniprot (https://www.uniprot.org/, accessed on 12 March 2021), the gene card database (https://www.genecards.org/, accessed on 12 March 2021) and selected references from the databases.

PROTEIN	DESCRIPTION	KEY WORDS
ANGPTL3	*Angiopoietin-related protein 3.* This gene encodes a member of a family of secreted proteins that function in angiogenesis. Binds to endothelial cells via integrin alpha-V/beta-3 (ITGAV:ITGB3), activates FAK, MAPK and Akt signaling pathways and induces cell adhesion and cell migration (PMID 11877390). Secreted from podocytes, may modulate properties of glomerular endothelial cells involving integrin alpha-V/beta-3 and Akt signaling (PMID 18535744). May increase the motility of podocytes. May induce actin filament rearrangements in podocytes implicating integrin alpha-V/beta-3 and Rac1 activation. Binds to hematopoietic stem cells (HSC) and is involved in the regulation of HSC activity probably implicating down-regulation of IKZF1/IKAROS (By similarity).	Angiogenesis<br>Integrin-mediated signaling pathways
APOA1	*Apolipoprotein A1*. This gene encodes the major protein component of high-density lipoprotein (HDL) in plasma. The encoded preproprotein is proteolytically processed to generate the mature protein, which promotes cholesterol efflux from tissues to the liver for excretion, and is a cofactor for lecithin cholesterolacyltransferase, an enzyme responsible for the formation of most plasma cholesteryl esters. Finally, APOA1 is important for induction of osteoblast differentiation (PMID 27359105).	Lipid metabolism<br>Osteoblast differentiation
APOE	*Apolipoprotein E*. The protein is a major apoprotein of the chylomicron and is essential for triglyceride and cholesterol metabolism. The encoded protein is important for osteoblast differentiation and bone healing (PMID 31534056, 27074899).	Lipid metabolism<br>Osteoblast differentiation
BCAM	*Basal cell adhesion molecule.* This glycoprotein is a member of the immunoglobulin superfamily and a receptor for the extracellular matrix protein laminin.	Extracellular matrix<br>Laminin receptor
CHI3L1	*Chitinase 3 like 1.* Chitinases catalyze the hydrolysis of chitin, and this gene encodes a glycoprotein member of the glycosyl hydrolase 18 family. The protein is secreted and is thought to play a role in tissue remodeling.<br>This secreted protein seems to be indirectly involved in the regulation of osteogenesis through effects on various cytokines and may also be important for osteoblastic differentiation though its effects on Notch signaling (PMID 28391634).	Glycosyl hydrolase<br>Tissue remodeling<br>Osteogenesis<br>Osteoblast differentiation
COL5A3	*Collagen type V alpha 3 chain.* This fibrillar collagen appears to regulate the assembly of heterotypic fibers composed of both type I and type V collagen.<br>Sp7/Osterix is an osteoblast-specific transcription factor that is important for osteoblast differentiation; this transcription factors increases Col5a3 transcription (PMID 20206127).	Extracellular matrix<br>Osteoblast differentiation
CRISPLD2	*Cysteine rich secretory protein LCCL domain containing 2.* The protein seems to be important for craniofacial development (PMID 17616516, 22887593).	Craniofacial development
CXCL12	*C-X-C motif chemokine ligand 12.* This stromal cell-derived chemokine is the ligand for the G-protein coupled receptor CXCR4.<br>The protein is important for maintaining musculoskeletal homeostasis (PMID 31336263), including osteogenic differentiation of MSCs (PMID 23935395). Disruption	Chemokine<br>Osteoblast differentiation<br>Osteopoerosis

	of CXCL12/CXCR4 signaling may have a role in the development of osteopoerosis (PMID 31336263, 32949783).	
DAAM2	*Dishevelled associated activator of morphogenesis 2.* WNT signals can be transduced to the RHOA signaling cascade through the Formin homology proteins DAAM1 and DAAM2 (PMID 16273260), and both WNT16 and DAAM2 are important for the predisposition to osteoporosis (PMID 30598549).	WNT<br>RhoA<br>Osteopoerosis
DMBT1	*Deleted in malignant brain tumors 1.* The encoded protein precursor is a glycoprotein containing multiple scavenger receptor cysteine-rich (SRCR) domains separated by SRCR-interspersed domains (SID). Animal studies suggest that the protein has a proangiogenic effect and protects against steroid-induced osteonecrosis (PMID 32447063).	Proangiogenic<br>Protect against osteonecrosis
ELN	*Elastin.* The encoded protein is one of the two components of elastic fibers that comprise part of the extracellular matrix. Degradation products of the encoded protein, known as elastin-derived peptides or elastokines, bind the elastin receptor complex. Elastokines can also contribute to cancer progression. Elastin is a marker of the osteogenic capacity of osteoblastic cells together with ALPL, COL1A2, DCN, ELN and RUNX2, and their effects are possibly mediated through regulation of TGF-ß1 pathway signaling (PMID 27711115, 24984278).<br><br>Elastin is involved in osteoblast mineralization of extracellular matrix (PMID 16817211).	Extracellular matrix<br>Osteoblast<br>Mineralization
F9	*Coagulation factor IX.* This vitamin K-dependent coagulation factor circulates as an inactive zymogen. This factor is converted to an active form by factor XIa, which excises the activation peptide and thus generates a heavy chain and a light chain held together by one or more disulfide bonds.<br>Clinical studies suggest excess of osteoporosis in patients with factor IX deficiency (hemophilia type B); direct effects of coagulation factors on osteoblasts/osteoblasts and/or indirect effects are possible mechanisms (PMID 29857920).	Coagulation<br>Osteoporosis
FBN2	*Fibrillin-2.* The encoded protein is a component of connective tissue microfibrils and may be involved in elastic fiber assembly. Fibrillin-2 control bone formation by regulating osteoblast differentiation through the differential modulation of endogenous TGFβ and bone morphogenetic protein signals, and through these mechanisms they become important regulators of bone remodeling (PMID 20729550). The protein seems important for bone mineralization and development of osteopoerosis (PMID 30690781), it is also important for TGFβ and BMP bioavailability during bone formation (PMID 20855508).	Extracellular matrix Mineralization<br>Osteoporosis<br>TGF-β1, BMP<br>Osteoblast differentiation
FGG	*Fibrinogen gamma chain.* The encoded protein is the gamma component of the fibrinogen coagulation factor.<br>The coagulation cascade may be important for regulation of bone mineralization, possibly through thrombin-mediated effects on osteoblasts (PMID 29857920).	Coagulation<br>Mineralization
HDAC7	*Histone deacetylase 7.* Histones play a critical role in transcriptional regulation and cell cycle progression.<br>HDAC7 suppresses osteoclastogenesis and attenuates β-catenin function (PMID 23204328). Furthermore, BMP2 stimulated nuclear export of HDAC7, and BMP2 signaling also regulates Runx2 activity via inhibition of HDAC7 transcriptional repression (PMID 19029091).	Transcription<br>Cell cycle<br>Osteoclast<br>BMP2<br>Runx2
IGFBP2	*Insulin like growth factor binding protein 2.* The protein encoded by this gene is one of six similar proteins that bind insulin-like growth factors (IGFs) I and II. The protein can be secreted into the bloodstream, where it binds IGF-I and IGF-II with high affinity, or it can remain intracellular, interacting with many different ligands. High expression levels of this protein promote the growth of several types of tumors and may be predictive of the chances of recovery of the patient. IGF is a main regulator of skeletal growth and	Skeletal growth<br>Osteoporosis

	decreased levels during aging seem important for the risk of demineralization/osteoporosis (PMID 29626053).	
JAG1	*Jagged canonical Notch ligand 1.* The jagged 1 protein encoded by JAG1 is the ligand for the receptor notch 1; the latter is involved in signaling processes.<br>Jag1 is important for osteoblast development and bone mineralization (PMID 29693255, 24491691).	Notch
LGALS9	*Galectin 9.* The galectins are a family of beta-galactoside-binding proteins implicated in modulating cell-cell and cell-matrix interactions. The protein encoded by this gene is an S-type lectin.<br>Galectin-9 induces osteoblast differentiation through the CD44/Smad signaling pathway in the absence of BMPs.	Extracellular matrix<br>Osteoblast differentiation<br>CD44-Smad
LIMS2/PINCH2	*LIM zinc finger domain containing 2.* This gene encodes a member of a small family of focal adhesion proteins which interacts with ILK (integrin-linked kinase), a protein which effects protein-protein interactions with the extracellular matrix.<br>The focal adhesion proteins Pinch1 and Pinch2/Lims2 regulate integrin activation and cell-extracellular matrix adhesion and migration; it is also important for bone homeostasis/mineralization (PMID 31723057).	Adhesion<br>Integrin<br>Mineralization
LRRC32	*Leucine rich repeat containing 32.* This gene encodes a type I membrane protein which contains 20 leucine-rich repeats. It is a TGFβ activating protein and may have growth enhancing and antiapoptotic effects in bone cells (PMID 33203838)	TGFβ
MFAP2	*Microfibrillar-associated protein 2.* This protein is a major part of elastin-associated microfibrils.<br>Microfibrillar-associated proteins (MFAPs) are extracellular matrix glycoproteins, which play a role in microfibril assembly, and MFAP2-deficient mice exhibit progressive osteopenia with elevated RANKL expression (PMID 32572962).	Extracellular matrix<br>Osteogenesis<br>RANKL
MUC5B	*Mucin 5B, oligomeric mucus/gel-forming.* This gene encodes a member of the mucin family of proteins, which are highly glycosylated macromolecular components. It can be expressed by chondrosarcoma (PMID 29138803).	Mucin
NTN4	Netrin 4. This gene encodes a member of the netrin family of proteins. Netrins are laminin-related proteins.<br>The protein inhibits osteoclastic differentiation and prevents bone loss in an animal model of osteoporosis (PMID 24846137)	Laminin<br>Mineralization<br>Anti-osteoporosis<br>Osteoclast
PSEN2	*Presenlin 2. The encoded protein is possibly* involved in the cleavage of the Notch receptor such that, they either directly regulate gamma-secretase activity, or themselves act are protease enzymes.<br>The protein is induced during osteoblastic differentiation together with other key regulators of Wnt, TGFβ and Notch signaling (PMID 12203032, 23600707). The protein is also involved in the regulation of autophagy and mitochondrial functions (PMID 30412492).	Notch<br>Osteoblast differentiation
PODN	*Podocan.* The protein is a member of the small leucine-rich repeat protein family and contains an amino terminal CX3CXCX7C cysteine-rich cluster followed by a leucine-rich repeat domain.<br>The protein can bind collagen (PMID 30519093, 15063725)	Extracellular matrix<br>Collagen
SPP1	*Secreted phosphoprotein 1/osteopontin.* The encoded protein is involved in the attachment of osteoclasts to the mineralized bone matrix. It is secreted and binds hydroxyapatite with high affinity. The osteoclast vitronectin receptor is found in the cell membrane and possibly important for this binding. This protein is also a cytokine that upregulates expression of interferon-γ and IL12.<br>SPP1 expression is regulated by Runx2 together with several other molecules involved in osteoblastic differentiation and functions. Runx2 is essential for osteoblast differentiation Its expression is upregulated in preosteoblasts, reaches the maximal level in immature osteoblasts, and is downregulated in mature osteoblasts. Runx2 enhances	Extracellular matrix<br>Osteoclast<br>Cytokine<br>RUNX2

	osteoblast progenitor proliferation progenitors by regulating *Fgfr2* and *Fgfr3 and through direct regulation of* hedgehog (*Ihh*, *Gli1*, and *Ptch1/Lims2*), Fgf (*Fgfr2* and *Fgfr3*), Wnt (*Tcf7*, *Wnt10b*, and *Wnt1*), and Pthlh (*Pthr1*) signaling pathway genes. Runx2 also induces the expression of major bone matrix protein genes, including *Spp1 together with Col1a1*, *Ibsp*, *Bglap2*, and *Fn1*.	
TFPI2	*Tissue factor pathway inhibitor 2.* This serine proteinase inhibitor inhibits a variety of serine proteases including factor VIIa/tissue factor, factor Xa, plasmin, trypsin, chymotryspin and plasma kallikrein.<br>The coagulation system is involved in bone homeostasis; the TFPI2 protein may interact with TGF/BMP signaling (PMID 19450457).	Proeinase inhibitor<br>Coagulation<br>TGF/BMP
TINAGL1	*Tubulointerstitial nephritis antigen like 1.* The protein is a secreted glycoprotein.<br>The encoded protein is regarded as a matrix protein, and animal models suggest that reduced function/level of Tinagl1 is associated with early craniofacial defects due to its Wnt-interacting effects (PMID 27243669).	Extracellular matrix<br>Wnt
TRAF6	• *TNF receptor-associated factor 6.* This gene encodes a member of the TNF receptor associated factor (TRAF) family of adaptor proteins that mediate signaling events from members of the TNF receptor and Toll/IL-1 receptor families to activate transcription factors such as NFκB and AP-1. Mice deficient in this protein exhibit osteopetrosis. This protein possesses ubiquitin ligase activity.<br>• Bone remodeling is a tightly coupled process consisting of repetitive cycles of bone resorption and formation. Both processes are governed by mechanical signals, including RANKL/RANK/OPG/TRAF6 signaling (PMID 17465692, 25368616).	TNF<br>NFκB<br>Ostepetrosis<br>Bone remodeling
VEGFC	• *Vascular endothelial growth factor C.* The encoded protein promotes angiogenesis and endothelial cell growth, it can affect vessel permeability and can activate VEGFR-2 and VEGFR-3 receptors.<br>• An animal model of osteoblast differentiation suggests that expression of the VEGF receptors (VEGFR1, VEGFR2, VEGF165R/neuropilin) coincided with expression of their ligands, being maximally expressed during mineralization. The expression profile of VEGFs/VEGF receptors suggests that they contribute to the regulation of bone remodeling by attracting endothelial cells and osteoclasts and by stimulating osteoblast differentiation (PMID 10803575).	Angiogenesis<br>Osteoblast differentiation<br>Bone remodelling

Table S3. Description of proteins expressed only in osteoblasts and included in the osteoblast-specific protein interaction network. The table gives a list (corresponding gene name, protein name) of those osteoblast-specific proteins that were included in the protein interaction networks presented in Figure 3. The keyword information is based on Uniprot (https://www.uniprot.org/), the gene card database (https://www.genecards.org/) and selected references from the databases.

PROTEIN	DESCRIPTION	KEY WORDS
APOA1	*Apolipoprotein A1*. This gene encodes the major protein component of high-density lipoprotein (HDL) in plasma. The encoded preproprotein is proteolytically processed to generate the mature protein, which promotes cholesterol efflux from tissues to the liver for excretion, and is a cofactor for lecithin cholesterol acyltransferase, an enzyme responsible for the formation of most plasma cholesteryl esters. Finally, APOA1 is important for induction of osteoblast differentiation (PMID 27359105).	Lipid metabolism
APOE	*Apolipoprotein E*. The protein is a major apoprotein of the chylomicron and is essential for triglyceride and cholesterol metabolism. The encoded protein is important for osteoblast differentiation and bone healing (PMID 31534056, 27074899).	Lipid metabolism
BRI3	*Brain protein I3*. Expressed in a wide range of organs. This molecular chaperon is a downstream target of Wnt signaling (PMID 20538055, 32555390).	Wnt<br>Chaperon
BST2	*Bone marrow stromal cell antigen 2.* The protein is important for osteoblast differentiation, this effect is mediated via BMP2 (PMID 27359105). The protein may also play a role in pre-B-cell growth.	Osteoblast differentiation<br>BMP2

B4GALT5	*Beta-1,4-galactosyltransferase 5.* This type II membrane-bound glycoprotein that appear to have exclusive specificity for UDP-galactose; it transfers galactose to similar acceptor sugars. It has an N-terminal hydrophobic signal sequence that directs the protein to the Golgi apparatus. Galactose administrations alters bone metabolism and leads to bone loss in animal models (PMID 32497865).	Galactose<br>Golgi<br>Metabolism<br>Bone loss
CD14	*CD14 molecule.* The encoded protein is a surface antigen that cooperates with other proteins to inhibit severe inflammatory responses. CD14 is a coreceptor for Toll like receptor 4 (TLR4), and these receptors are involved in the regulation of osteoblast metabolism and bone mineral density as well as development of inflammation-associated osteopoerosis (PMID 29867550).	Osteoblast<br>TLR4<br>Mineralization
CD36	*CD36 molecule.* The encoded protein serves as a receptor for thrombospondin, widely distributed proteins involved in adhesive processes. CD36 binds to collagen, thrombospondin and phospholipids. It binds long chain fatty acids and may function in the transport and/or as a regulator of fatty acid transport.	Adhesion Thrombospondin<br>Collagen<br>Fatty acid
CHI3L1	*Chitinase 3 like 1.* Chitinases catalyze the hydrolysis of chitin, and this gene encodes a glycoprotein member of the glycosyl hydrolase 18 family. The protein is secreted and is thought to play a role in tissue remodeling.<br>This chitinase is a marker of MSC differentiation, including differentiation in the direction of mature mesenchymal phenotypes, including osteocytes (PMID 23671604). It supports angiogenesis possibly through stimulation of proinflammatory and proangiogenic cytokines/chemokines (e.g. CCL2, CXCL2) (PMID 26733160).	Glycosyl hydrolase<br>Tissue remodeling<br>Angiogenesis<br>Osteocyte differentiation<br>Chemokine
CHMP1B	*Charged multivesicular body protein 1B.* CHMP1B belongs to the chromatin-modifying protein/charged multivesicular body protein (CHMP) family. These proteins are components of ESCRT-III (endosomal sorting complex required for transport III), a complex involved in degradation of surface receptor proteins and formation of endocytic multivesicular bodies. Some CHMPs have both nuclear and cytoplasmic/vesicular distributions, and CHMP1A is required for regulation of cell cycle progression.<br>ESCRT-III associated proteins (e.g. CHMP1B) are important for intracellular transport and receptor trafficking, including BMP receptors (PMID 31587092).	Protein degradation<br>Chromatin<br>Cell cycle<br>Receptor trafficking<br>BMP receptors
CHMP2B	*Charged multivesicular body protein 2B.* This component of the heteromeric ESCRT-III complex (Endosomal Sorting Complex Required for Transport III) that functions in the recycling or degradation of cell surface receptors. ESCRT-III functions in the concentration and invagination of ubiquitinated endosomal cargos into intralumenal vesicles.<br>ESCRT-III associated proteins are important for intracellular transport and receptor trafficking, including BMP receptors (PMID 31587092).	Ubiquitin<br>Endosome<br>Receptor trafficking
ELN	*Elastin.* The encoded protein is one of the two components of elastic fibers that comprise part of the extracellular matrix. Degradation products of the encoded protein, known as elastin-derived peptides or elastokines, bind the elastin receptor complex. Elastokines can also contribute to cancer progression.<br>Elastin is a marker of the osteogenic capacity of osteoblastic cells together with ALPL, COL1A2, DCN, ELN and RUNX2, and their effects are possibly mediated through regulation of TGF-ß1 pathway signaling (PMID 27711115, 24984278).<br><br>Elastin is involved in osteoblast mineralization of extracellular matrix (PMID 16817211).	Extracellular matrix<br>Osteogenesis<br>TGF-ß1
ENTPD1/CD39	*Ectonucleoside triphosphate diphosphohydrolase 1.* The protein encoded is a plasma membrane protein that hydrolyzes extracellular ATP and ADP to AMP.<br>CD39/ENTPD1 drives the sequential hydrolysis of both adenosine triphosphate (ATP) and adenosine diphosphate (ADP) to adenosine monophosphate (AMP). AMP can be further degraded by CD73/ecto-5′-nucleotidase, to adenosine. (PMID 17502665).	Plasma membrane<br>ATP/ADP hydrolysis

EPHA4	*EPH receptor A4.* This gene belongs to the ephrin receptor subfamily of the protein-tyrosine kinase family. EPH and EPH-related receptors have been implicated in mediating developmental events. The ephrin receptors are divided into 2 groups based on the similarity of their extracellular domain sequences and their affinities for binding ephrin-A and ephrin-B ligands. Multiple transcript variants encoding different isoforms have been found for this gene.<br>See EPHB4 for a detailed comment.	Ephrine receptor
EPHB4	*EPH receptor B4.* Ephrin receptors and their ligands, the ephrins, mediate numerous developmental processes. Based on their structures and sequence relationships, ephrins are divided into the ephrin-A (EFNA) class, which are anchored to the membrane by a glycosylphosphatidylinositol linkage, and the ephrin-B (EFNB) class, which are transmembrane proteins. The Eph family of receptors are divided into 2 groups based on the similarity of their extracellular domain sequences and their affinities for binding ephrin-A and ephrin-B ligands. Ephrin receptors make up the largest subgroup of the receptor tyrosine kinase (RTK) family. The protein encoded by this gene binds to ephrin-B2.<br>EphA2 was expressed in osteoclast precursors and osteoblasts (PMID19299512). Bones cannot be properly formed or maintained without cell-cell interactions through ephrin ligands and Eph receptors; EphA2 is involved in the regulation of osteoblastic bone formation, mineralization and remodeling (PMID 22660185, 21724962, 25604255). Osteoblasts stimulated with ephrin-A2 in vitro significantly altered osteoblast gene expression including expression of RUNX2, ALPL and ALP (PMID 21724962). Furthermore, the bidirectional ephrin/receptor signaling then regulates bone remodeling at the initiation phase, and these effects of EphA2 are possibly mediated by RhoA (PMID 19299512). Finally, Local exosome-mediated communication between cells has an important role in bone homeostasis, and exosomes can specifically recognize osteoblasts through the interaction between ephrinA2 and EphA2 (PMID 27462462).	Ephrine receptor<br>Bone remodeling<br>Osteoblast<br>Mineralization<br>RUNX2<br>RhoA<br>Exosomes
FAM20A	*FAM20A Golgi associated secretory pathway pseudokinase.* This protein is likely secreted and may function in hematopoiesis.<br>The family with sequence similarity 20 (Fam20) kinases phosphorylate extracellular substrates and play important roles in biomineralization (PMID 23754375). Fam20C is the Golgi casein kinase that phosphorylates secretory pathway proteins within Ser-x-Glu/pSer motifs. Mutations in Fam20C cause Raine syndrome, an osteosclerotic bone dysplasia (PMID 23754375), and FAM20A binds to and regulates FAM20C localization (PMID 27292199).	Golgi<br>Pseudokinase<br>Mineralization<br>Golgi casein kinase
FBN2	*Fibrillin-2.* The encoded protein is a component of connective tissue microfibrils and may be involved in elastic fiber assembly. Fibrillin-2 control bone formation by regulating osteoblast differentiation through the differential modulation of endogenous TGFβ and bone morphogenetic protein signals, and through these mechanisms they become important regulators of bone remodeling (PMID 20729550). The protein seems important for bone mineralization and development of osteopoerosis (PMID 30690781), it is also important for TGFβ and BMP bioavailability during bone formation (PMID 20855508).	Extracellular matrix<br>Mineralization<br>Osteoporosis<br>TGF-ß1, BMP<br>Osteoblast differentiation
FGG	*Fibrinogen gamma chain.* The encoded protein is the gamma component of the fibrinogen coagulation factor.<br>The coagulation cascade may be important for regulation of bone mineralization, possibly through thrombin-mediated effects on osteoblasts (PMID 29857920).	Mineralization
FYN	*FYN proto-oncogene, Src family tyrosine kinase.* This gene encodes a membrane-associated tyrosine kinase that has been implicated in the control of cell growth. The protein associates with the p85 subunit of phosphatidylinositol 3-kinase and interacts with the fyn-binding protein.	Osteoblast differentiation

	An experimental study suggests that Lyn, Fyn, and FGFR2 interacted with the ubiquitin ligase c-Cbl and ubiquitin in the regulation of osteoblast differentiation (PMID 15190072).	
GUSB	*Glucuronidase beta.* This gene encodes a hydrolase that degrades glycosaminoglycans, including heparan sulfate, dermatan sulfate, and chondroitin-4,6-sulfate. The enzyme forms a homotetramer that is localized to the lysosome.	Lysosome
HLA-A<br>HLA-B<br>HLA-C<br>HLA-DRB1<br>HLA-F	HLA molecules are important in the regulation of both innate and adaptive immunity.	Immunoregulation<br>Inflammation
KCNAB2	*Potassium voltage-gated channel subfamily A regulatory beta subunit 2.* Voltage-gated potassium channels represent the most complex class of voltage-gated ion channels. This gene encodes a member of the potassium channel, voltage-gated, shaker-related subfamily.	Potassium channel
KRT77	*Keratin 77.* Keratins are intermediate filament proteins responsible for the structural integrity of epithelial cells and are subdivided into epithelial keratins and hair keratins. This gene encodes an epithelial keratin. Keratins are parts of the cytoskeleton (PMID 15495250, 21893596).	Keratin<br>Cytoskeleton
KRT80	*Keratin 80.* This gene's expression profile shows that it encodes a type II epithelial keratin, although structurally the encoded protein is more like a type II hair keratin. This protein is involved in cell differentiation. Keratins are parts of the cytoskeleton (PMID 15495250, 21893596),	Keratin<br>Cytoskeleton
LMNA	*Laminin A/C.* The nuclear lamina consists of a two-dimensional matrix of proteins located next to the inner nuclear membrane. The lamin family of proteins make up the matrix. Lamin proteins are thought to be involved in nuclear stability, chromatin structure and gene expression.<br>Lamin A/C is fundamental for and differentiation of MSCs that are progenitors of osteoblasts; laminins thereby become important for bone homeostasis (PMID 32466483).	Nuclear envelope<br>Transcription<br>Differentiation<br>Bone homeostasis
LYZ	*Lysozyme. T*his gene encodes human lysozyme. The protein may be a modulator and adaptor of Mucin 5B (PMID 12568489).	Mucin 5B
MAP3K7	*Mitogen-activated protein kinase 7.* The protein is a member of the serine/threonine protein kinase family. This kinase mediates the signaling transduction induced by TGFβ and BMP and controls a variety of cell functions including transcription regulation and apoptosis. In response to IL-1, this protein forms a kinase complex including TRAF6, MAP3K7P1/TAB1 and MAP3K7P2/TAB2; this complex is required for the activation of NFκB. This kinase can also activate MAPK8/JNK, MAP2K4/MKK4.<br>The protein is probably a modulator of osteoblastic differentiation (PMID 29753717).	Kinase<br>Signal transduction<br>TGF<br>BMP<br>TRAF6
MFAP2	*Microfibrillar-associated protein 2.* This protein is a major part of elastin-associated microfibrils.<br>Microfibrillar-associated proteins (MFAPs) are extracellular matrix glycoproteins, which play a role in microfibril assembly, and MFAP2-deficient mice exhibit progressive osteopenia with elevated RANKL expression (PMID 32572962).	Extracellular matrix<br>Osteogenesis<br>RANKL
MUC5B	*Mucin 5B, oligomeric mucus/gel-forming.* This gene encodes a member of the mucin family of proteins, which are highly glycosylated macromolecular components. It can be expressed by chondrosarcoma (PMID 29138803).	Mucin<br>Lysozyme
NTPCR	*Nucleoside-triphosphatase, cancer-related.* The protein is a non-specific nucleoside triphosphatase.	Nucleoside
PIGR	*Polymeric immunoglobulin receptor.* This gene is a member of the immunoglobulin superfamily. The encoded poly-Ig receptor binds polymeric immunoglobulin molecules and is secreted.	Protein secretion

PRKCQ	*Protein kinase C theta.* Protein kinase C (PKC) is a family of serine- and threonine-specific protein kinases that can be activated by calcium and the second messenger diacylglycerol. Each member of the PKC family has a specific expression profile This protein is a calcium-independent and phospholipid-dependent protein kinase that is required for activation of NFκB and AP-1.<br>A previous study suggests that PKC βII together with and PKC δ/θ mediate BMP-4-induced osteoblastic differentiation (PMID 20971075).	NFκB<br>AP-1<br>Osteoblast differentiation
PRUNE2	*Prune homolog 2 with BCH domain.* The encoded protein has several roles, including suppression of RhoA activity. A high molecular weight isoform colocalizes with Adaptor protein complex 2, beta-Adaptin and endodermal markers, suggesting an involvement in post-endocytic trafficking.<br>Notch signaling seems to upregulate the transcription of PRUNE2 (PMID 28300657). Previous studies suggest that this molecule is involved in regulation of cell differentiation and apoptosis (PMID 22710163).	RhoA<br>Notch<br>Differentiation<br>Apoptosis
PSEN2	*Presenlin 2. The encoded protein is possibly* involved in the cleavage of the Notch receptor such that, they either directly regulate gamma-secretase activity, or themselves act are protease enzymes.<br>The protein is induced during osteoblastic differentiation together with other key regulators of Wnt, TGFβ and Notch signaling (PMID 12203032, 23600707). The protein is also involved in the regulation of autophagy and mitochondrial functions (PMID 30412492).	Notch<br>Osteoblast differentiation
SPP1	*Secreted phosphoprotein 1/osteopontin.* The encoded protein is involved in the attachment of osteoclasts to the mineralized bone matrix. It is secreted and binds hydroxyapatite. The osteoclast vitronectin receptor may be involved in the binding to this protein. This protein is also a cytokine that upregulates expression of interferon-gamma and interleukin-12.<br>RUNX2 induces the expression of this gene during osteoblastic differentiation (PMID 30987410).	RUNX2<br>Extracellular release<br>Osteoclast
TANK	*TRAF family member associated NFKB activator.* The TRAF (tumor necrosis factor receptor-associated factor) protease family associate with and transduce signals from members of the TNF superfamily. The protein encoded by this gene is found in the cytoplasm and can bind to TRAF1, TRAF2, or TRAF3, thereby inhibiting TRAF function by sequestering the TRAFs in a latent state in the cytoplasm and can inhibit NFκB activation.<br>The protein is induced during osteoblast differentiation; it inhibits NFκB through inhibition of TRAF6 activation, is a negative regulator osteoclastogenesis and is thereby important for bone remodeling (PMID 22773835).	Protease<br>TRAF6 inhibition<br>NFκB<br>Bone remodeling
TRAF6	• *TNF receptor-associated factor 6.* This gene encodes a member of the TNF receptor associated factor (TRAF) family of adaptor proteins that mediate signaling events from members of the TNF receptor and Toll/IL-1 receptor families to activate transcription factors such as NFκB and AP-1. Mice deficient in this protein exhibit osteopetrosis. This protein possesses ubiquitin ligase activity.<br>• Bone remodeling is a tightly coupled process consisting of repetitive cycles of bone resorption and formation. Both processes are governed by mechanical signals, including RANKL/RANK/OPG/TRAF6 signaling (PMID 17465692, 25368616).	TNF<br>NFκB<br>Osteopetrosis<br>Bone remodeling<br>MAP3K7
VEGFC	• *Vascular endothelial growth factor C.* The encoded protein promotes angiogenesis and endothelial cell growth, it can affect vessel permeability and can activate VEGFR-2 and VEGFR-3 receptors.<br>• An animal model of osteoblast differentiation suggests that expression of the VEGF receptors (VEGFR1, VEGFR2, VEGF165R/neuropilin) coincided with expression of their ligands, being maximally expressed during mineralization. The	Angiogenesis<br>Osteoblast differentiation<br>Bone remodelling

expression profile of VEGFs/VEGF receptors suggests that they contribute to the regulation of bone remodeling by attracting endothelial cells and osteoclasts and by stimulating osteoblast differentiation (PMID 10803575).

Table S4. Proteins expressed only in MSCs. The table gives a list (corresponding gene name, protein name) of all proteins that could be quantified in MSCs but were not detected for any of the osteoblast donors.

AASS	Alpha-aminoadipic semialdehyde synthase, mitochondrial;Lysine ketoglutarate reductase;Saccharopine dehydrogenase
ACTR6	Actin-related protein 6
ALAS1	5-aminolevulinate synthase, nonspecific, mitochondrial
AMOTL1	Angiomotin-like protein 1
AP1B1	AP-1 complex subunit beta-1
ARID3A	AT-rich interactive domain-containing protein 3A
ATR	Serine/threonine-protein kinase ATR
BIN3	Bridging integrator 3
BRD9	Bromodomain-containing protein 9
BRMS1L	Breast cancer metastasis-suppressor 1-like protein
C19orf47	Uncharacterized protein C19orf47
C1QTNF3	Complement C1q tumor necrosis factor-related protein 3
C5orf30	UNC119-binding protein C5orf30
C7orf55;LUC7L2	UPF0562 protein C7orf55;Putative RNA-binding protein Luc7-like 2
CBX6;CBX8	Chromobox protein homolog 6;Chromobox protein homolog 8
CCDC115	Coiled-coil domain-containing protein 115
CDK17;CDK16	Cyclin-dependent kinase 17;Cyclin-dependent kinase 16
CENPV	Centromere protein V
CEP41	Centrosomal protein of 41 kDa
CEPT1	Choline/ethanolaminephosphotransferase 1
CHPT1	Cholinephosphotransferase 1
CLNS1A	Methylosome subunit pICln
COQ9	Ubiquinone biosynthesis protein COQ9, mitochondrial
CPNE7	Copine-7
CSNK1D	Casein kinase I isoform delta
CYB561D2	Cytochrome b561 domain-containing protein 2
DCAF7	DDB1- and CUL4-associated factor 7
EPB41L5	Band 4.1-like protein 5
FAM96A	MIP18 family protein FAM96A
FBLN2	Fibulin-2
FBXW9	F-box/WD repeat-containing protein 9
FLII	Protein flightless-1 homolog
FOXK2	Forkhead box protein K2
GDF15	Growth/differentiation factor 15
GTF2E2	Transcription initiation factor IIE subunit beta
HAUS1	HAUS augmin-like complex subunit 1
HCFC2	Host cell factor 2
HPDL	4-hydroxyphenylpyruvate dioxygenase-like protein
IER3IP1	Immediate early response 3-interacting protein 1
INO80C	INO80 complex subunit C
INTS5	Integrator complex subunit 5
KANK1	KN motif and ankyrin repeat domain-containing protein 1
KCTD21	BTB/POZ domain-containing protein KCTD21

KLHDC4	Kelch domain-containing protein 4
KRT82	Keratin, type II cuticular Hb2
LCMT2	tRNA wybutosine-synthesizing protein 4
LRWD1	Leucine-rich repeat and WD repeat-containing protein 1
LYPLA2	Acyl-protein thioesterase 2
MAT2B	Methionine adenosyltransferase 2 subunit beta
METTL2A	Methyltransferase-like protein 2A
MEX3C	RNA-binding E3 ubiquitin-protein ligase MEX3C
MFSD5	Molybdate-anion transporter
MGAT5	Alpha-1,6-mannosylglycoprotein 6-beta-N-acetylglucosaminyltransferase A
MIER1	Mesoderm induction early response protein 1
MKL2	MKL/myocardin-like protein 2
MLLT11	Protein AF1q
MORC2	MORC family CW-type zinc finger protein 2
MRPL53	39S ribosomal protein L53, mitochondrial
MTG1	Mitochondrial ribosome-associated GTPase 1
MT-ND5	NADH-ubiquinone oxidoreductase chain 5
MYO10	Unconventional myosin-X
MYO1B	Unconventional myosin-Ib
NACAP1	Putative nascent polypeptide-associated complex subunit alpha-like protein
NHSL1	NHS-like protein 1
NOP10	H/ACA ribonucleoprotein complex subunit 3
NRG1	Pro-neuregulin-1, membrane-bound isoform;Neuregulin-1
NRM	Nurim
NSMCE2	E3 SUMO-protein ligase NSE2
NT5C3B	7-methylguanosine phosphate-specific 5-nucleotidase
OSTM1	Osteopetrosis-associated transmembrane protein 1
PCDHGB5	Protocadherin gamma-B5
PDCD1LG2	Programmed cell death 1 ligand 2
PINX1	PIN2/TERF1-interacting telomerase inhibitor 1
PIP5K1A	Phosphatidylinositol 4-phosphate 5-kinase type-1 alpha
PMEPA1	Protein TMEPAI
POLR3D	DNA-directed RNA polymerase III subunit RPC4
PRIM2	DNA primase large subunit
PRPF18	Pre-mRNA-splicing factor 18
PTPMT1	Phosphatidylglycerophosphatase and protein-tyrosine phosphatase 1
RAB6B	Ras-related protein Rab-6B
RC3H1	Roquin-1
SAP130	Histone deacetylase complex subunit SAP130
SEPN1	Selenoprotein N
SESN2	Sestrin-2
SFXN4	Sideroflexin-4
SH3BP5L	SH3 domain-binding protein 5-like
SH3PXD2A	SH3 and PX domain-containing protein 2A
SHKBP1	SH3KBP1-binding protein 1
SLC16A6	Monocarboxylate transporter 7
SLC2A10	Solute carrier family 2, facilitated glucose transporter member 10
SLC6A9	Sodium- and chloride-dependent glycine transporter 1
SMAD1	Mothers against decapentaplegic homolog 1

SMARCD3	SWI/SNF-related matrix-associated actin-dependent regulator of chromatin subfamily D member 3
SMPDL3A	Acid sphingomyelinase-like phosphodiesterase 3a
SRRD	SRR1-like protein
SYNJ2BP	Synaptojanin-2-binding protein
TBX15	T-box transcription factor TBX15
TBX3	T-box transcription factor TBX3
TCF12	Transcription factor 12
TFB1M	Dimethyladenosine transferase 1, mitochondrial
TIMM17B	Mitochondrial import inner membrane translocase subunit Tim17-B
TMED8	Protein TMED8
TNFAIP8L3	Tumor necrosis factor alpha-induced protein 8-like protein 3
TOX2	TOX high mobility group box family member 2
UBE2T	Ubiquitin-conjugating enzyme E2 T
USP36	Ubiquitin carboxyl-terminal hydrolase 36
VPRBP	Protein VPRBP
WASF1	Wiskott-Aldrich syndrome protein family member 1
YAF2	YY1-associated factor 2
ZMAT2	Zinc finger matrin-type protein 2
ZNF280C	Zinc finger protein 280C

Table S5. Proteins involved in differentiation/development of osteoblasts and showing significant differences when comparing osteoblasts with MSCs. Only proteins with detectable levels both for osteoblasts and MSCs for at least three donors were included in the statistical comparison. Based on the QuickGO database 60 proteins involved in differentiation/development of osteoblasts have been identified, and 11 of these proteins could be quantified in our present study and showed significant differences between osteoblasts and MSCs. Three additional proteins showing significant differences were identified based on the Uniprot database when using a similar strategy. The 14 proteins are listed below (corresponding gene names) together with the protein name and a brief description of the protein together with keywords describing the most important/relevant biological functions. The data presented in the description/key word sections are based on information from the gene card database together with selected references from the gene database.

Protein	Description	Keywords
CEBPB	*CCAAT enhancer binding protein beta*. This gene encodes a transcription factor. The protein seems to be important for the regulation/balance of osteogenic versus adipogenic differentiation (PMID 28240601).	Transcription<br>Osteoblast differentiation
CYR61 (CCN1)	*CCN family member 1/cellular communication network factor 1.* This secreted protein is growth factor-inducible and promotes adhesion. It interacts with several integrins and with sulfate proteoglycans. This protein also plays a role in cell proliferation, differentiation, angiogenesis, apoptosis and extracellular matrix formation.<br>This matricellular protein serves regulatory rather than structural roles. It is found in mineralized tissues and influences bone healing *in vivo* and osteogenic differentiation *in vitro*. It exerts its effects on mature osteoblasts/osteocytes to modulate the bone mass and can alter the expression of the genes RANKL, VEGFA and SOST. The increased SOST expression is important for regulation of the bone mass through its effects on Wnt signaling; additional angiogenic effects are also important for CYR61 effects on bone mass (PMID 29351359). CYR61/CCN1 regulates the expression of PTH1R through interaction with the $\alpha V\beta 3$ and/or $\alpha V\beta 5$ integrins; the $\alpha V\beta 3/\beta 5$-	Osteoblast differentiation<br>Extracellular matrix<br>Angiogenic<br>Adhesion<br>Integrins<br>Wnt<br>PTH<br>BMP2

	binding domain of CCN1 is required for the PTH associated anabolic activity in bone cells (PMID 32634285). CYR61 also up-regulates BMP-2 protein expression, resulting in increased proliferation and osteoblastic differentiation through activation of $\alpha V\beta 3$ integrin/integrin-linked kinase/ERK signaling (PMID 20675382)<br>Gene expression profiling of Wnt3A stimulated MSCs showed increased expression of 220 genes, including CYR61 together with other members of the CNN family (CCN2/connective tissue growth factor, CCN5/WISP2). CCN1/Cyr61 was then upregulated at the early stage of Wnt3A stimulation. It is a direct target of canonical Wnt/β-catenin signaling and seems to have an important role in Wnt3A-induced osteoblast differentiation from MSCs (PMID 16581771).<br>Finally, CYR61 is an inhibitor of osteoclast formation; given that CYR61 also stimulates osteoblasts it may therefore be a bifunctional local regulator of bone remodeling (PMID 17823253)	
DDX21	*DExD-box helicase 21.* DEAD box proteins are putative RNA helicases. This protein unwinds double-stranded RNA, folds single-stranded RNA, and may play important roles in ribosomal RNA biogenesis, RNA editing, RNA transport, and general transcription.<br>The protein has cell type specific effects; it is important in regulation of hematopoiesis, but it is not known whether the effect on hematopoiesis/bone marrow cells involve or influence osteoblasts or other mesenchymal cells (PMID 29364875).	RNA helicase<br>Ribosome<br>Transcription
ENPP1	*Ectonucleotide pyrophosphatase/phosphodiesterase 1.* This gene is a member of the ecto-nucleotide pyrophosphatase-phosphodiesterase family. The encoded protein is a type II transmembrane glycoprotein, it has broad specificity and cleaves several substrates, including phosphodiester pyrophosphate bonds of nucleotides and nucleotide sugars.<br>This protein is important for the regulation of osteoblastic functions and phosphate metabolism/transport, the formation of matrix vesicles and bone mineralization (PMID 29411103). It may also me involved in osteoblastic differentiation (PMID 30467547).	Membrane protein<br>Nucleotide cleavage<br>Phosphate and phosphodiester bonds<br>Bone mineralization<br>Differentiation
EPHA2	*Ephrin type-A receptor 2.* This protein belongs to the ephrin receptor subfamily of the protein-tyrosine kinase family. Receptors in the EPH subfamily typically have a single kinase domain and an extracellular region containing a Cys-rich domain and 2 fibronectin type III repeats. The ephrin receptors are divided into 2 groups based on the similarity of their extracellular domain sequences and their affinities for binding ephrin-A and ephrin-B ligands. This gene encodes a protein that binds ephrin-A ligands.<br>EphA2 was expressed in osteoclast precursors and osteoblasts (PMID19299512). Bones cannot be properly formed or maintained without cell-cell interactions through ephrin ligands and Eph receptors; EphA2 is involved in the regulation of osteoblastic bone formation, mineralization and remodelling	Tyrosine kinase<br>Transcription<br>RhoA<br>Exosome

	(PMID 22660185, 21724962, 25604255). Osteoblasts stimulated with ephrin-A2 in vitro significantly altered osteoblast gene expression including expression of RUNX2, ALPL and ALP (PMID 21724962).<br>The bidirectional ephrin/receptor signaling then regulates bone remodeling at the initiation phase, and these effects of EphA2 are possibly mediated by RhoA (PMID 19299512).<br>Local exosome-mediated communication between cells has an important role in bone homeostasis, and exosomes can specifically recognize osteoblasts through the interaction between ephrinA2 and EphA2 (PMID 27462462).	
FASN	*Fatty acid synthase.* The enzyme is a multifunctional protein. Its main function is to catalyze the synthesis of palmitate from acetyl-CoA and malonyl-CoA, in the presence of NADPH, into long-chain saturated fatty acids. Triglyceride metabolism and its regulation by this enzyme seem important in osteoblastic differentiation (PMID 23588618, 23588618).	Triglyceride Metabolism
FBN1	*Fibrillin-1.* The encoded preproprotein is proteolytically processed to generate two proteins including the extracellular matrix component fibrillin-1 and the protein hormone asprosin. Fibrillin-1 is an extracellular matrix glycoprotein that serves as a structural component of calcium-binding microfibrils. These microfibrils provide force-bearing structural support in elastic and nonelastic connective tissue throughout the body. Asprosin regulates glucose homeostasis.<br>FBN1 is a structural component of bone and is constitutively expressed by osteoblasts (PMID 10865210). It differentially regulates TGF-β and BMP bioavailability in bone and thereby bone formation (PMID 20855508); this seems to be at least partly mediated by altered Notch signaling in osteoblasts (PMID 21538048).	Extracellular matrix<br>Bone Mineralization<br>TGFβ<br>BMP<br>Notch
FHL2	*Four and a half lim domain 2.* This protein is a member of the four-and-a-half-LIM-only protein family.<br>FHL2 interacts with many integrins and transcription factors and is important in osteoblast differentiation. Overexpression of FHL2 increases the accumulation of osteoblast differentiation markers and matrix mineralization. Integrin-matrix interaction plays a critical role in osteoblast function possibly through interactions with actin-binding proteins. FHL2 co-localizes with $\alpha V\beta 5$ integrins at the focal adhesion sites. Osteoblasts overexpressing FHL2 exhibited increased adhesion to and migration on matrix proteins, and its stimulation of CREB activity is also dependent on integrins. Furthermore, the expression of osteoblast differentiation markers and Msx2 is upregulated and bone matrix mineralization is increased by FHL2 overexpression. Finally, However, FHL2 is also present in nuclei (PMID 16355270).<br>Wnt signaling is an important pathway that controls the osteogenic differentiation of MSCs, and FHL2 controls MSC osteogenic differentiation as well as bone formation through its cooperation with Wnt/β-catenin signaling (PMID 23201222).	Integrin<br>Mineralization<br>Adhesion<br>Migration<br>Wnt

GPNMB (osteoactin)	*Glycoprotein Nmb*. The encoded protein is a type I transmembrane glycoprotein also referred to as osteoactivin. GPNMB RNA and protein expression increases during active bone regeneration post-fracture and high expression is seen especially during osteoblast differentiation (PMID 25899717, 24462663). It is expressed in several tissues but is most highly expressed in bone compared with non-osseous tissues (PMID 11746512). The highest osteoblast levels are observed during the later stages of bone matrix production/maturation and mineralization; the expression is correlated with expression of alkaline phosphatase and osteocalcin. Its expression in bone is regarded as osteoblast-specific (PMID 11746512), and MiR-150 is another regulator of GPNMB expression (PMID 32526406).	Extracellular matrix<br>Mineralization<br>ALP
GTPBP4	*GTP binding protein 4.* The GTP-binding proteins are GTPases and function as molecular switches between the active GTP-bound and the GDP-bound state. When an extracellular ligand binds to a G-protein-linked receptor, the receptor changes its conformation to replace GDP with GTP. GTPBP4 can reorganized actin cytoskeleton through reduced RhoA activity (PMID 27720713); Rho GTPase and Wnt signaling have critical roles in bone cell physiology (PMID: 23856044).	GTPase<br>Actin<br>RhoA<br>Wnt
ITGA11	*Integrin subunit alpha 11.* This gene encodes an alpha integrin that binds collagen. Furthermore, Osteolectin/Clec11a is an osteogenic growth factor required to maintain the skeletal bone mass. It acts on osteogenic progenitors to promote their differentiation into osteoblasts. Integrin α11 is an Osteolectin receptor, and ligation of integrin $\alpha 11\beta 1$ activates the Wnt pathway (PMID 30632962).	Integrin<br>Collagen<br>Osteolectin<br>Clec11a<br>Wnt
LOX	*Lysyl oxidase.* This protein is a member of the lysyl oxidase family. Alternative splicing results in multiple transcript variants; at least one of them encodes a preprotein that is proteolytically processed to generate a regulatory propeptide and the mature enzyme. Its copper-dependent amine oxidase activity functions in the crosslinking of collagens and elastin. Collagen cross-linking is regulated by lysyl oxidase. LOX can also influence bone remodeling through effects both on osteoblast and osteoclast activity (PMID 32165300) and is thereby important for maintenance of normal bone turnover (PMID 26627907). LOX-mediated organization of collagen fibers in the extracellular matrix seems to be an important regulator of osteoblastogenesis (PMID 26497171). Finally, LOX is important for mineral nodule formation and osteoblastic differentiation, i.e. expression of the osteoblastic markers type I collagen, bone sialoprotein and Runx2 (PMID 19458888).	Extracellular matrix<br>Collagen<br>Elastin<br>Bone turnover<br>Osteoblast<br>Osteoclast<br>Runx2
TMEM119	*Transmembrane protein 119.* TNMEM119 expression is rapidly induced by PTH and it increases with time in MSCs committed to the osteoblast lineage by BMP-2. Overexpression of TMEM119 was associated with increased levels of Runx2, osteocalcin, alkaline phosphatase, and β-catenin. It is regarded as an important molecule in the pathway downstream of PTH	PTH<br>BMP2<br>Runx2<br>ALP<br>Osteocalcin<br>Wnt/catenin

	and Smad3 signaling in osteoblasts (PMID 21239498, 24362451).	
TPM4	• *Tropomyosin 4.* This member of the tropomyosin family of actin-binding proteins modulates the cytoskeleton of non-muscle cells. It is a dimer of coiled-coil proteins that polymerize end-to-end along the major groove in most actin filaments. The protein provides stability to the filaments and regulates the access of other actin-binding proteins. The protein seems important for bone remodeling/mineralization (PMID 30739146).	Cytoskeleton<br>Actin<br>Mineralization

Table S6. Proteins showing a significant difference between male and female osteoblast donors. For each protein, the table presents the corresponding gene name, protein name, fold change from the comparison of males/females (log_2 transformed), the *p*-value and the *z*-score.

Gene Name	Protein Name	Fold Change Male/Female	*p*-value	*z*-score
APOE	Apolipoprotein E	−1,08	0.03467809	0,01676925
ATP6V1F	V-type proton ATPase subunit F	−1,44	0.0431466	0,00212157
BPGM	Bisphosphoglycerate mutase	0,99	0.02434383	0,00833979
C1orf198	Uncharacterized protein C1orf198	1,08	0.00930514	0,00488407
C8orf82	UPF0598 protein C8orf82	1,01	0.02221794	0.00756661
−CHAMP1	Chromosome alignment-maintaining phosphoprotein 1	−0.95	0.03732206	0.03120167
CNN1	Calponin-1	3.48	0.04878159	0
COMMD9	COMM domain-containing protein 9	−0.88	0.04879125	0.04173354
CPNE2	Copine-2	−1.33	0.00877175	0.00405265
DCPS	m7GpppX diphosphatase	−0.99	0.02901125	0.02516671
DIAPH2	Protein diaphanous homolog 2	1.23	0.03345499	0.00156539
EIF2B1	Translation initiation factor eIF-2B subunit alpha	0.88	0.01190378	0.01687002
FAM129A	Protein Niban	−1.31	0.02631683	0.00459538
FAM195B	Protein FAM195B	1.27	0.04580781	0.00117364
FRG1	Protein FRG1	1.01	0.0414877	0.00760812
GPX1	Glutathione peroxidase 1	−2.05	0.02552418	1.9318E-05
IVNS1ABP	Influenza virus NS1A-binding protein	−0.87	0.00559874	0.04442645
LTBP1	Latent-transforming growth factor beta-binding protein 1	−1.30	0.04820586	0.00487305
MEST	Mesoderm-specific transcript homolog protein	1.44	0.00437263	0.0002999
METTL7A	Methyltransferase-like protein 7A	1.31	0.02646207	0.00089613
MIB1	E3 ubiquitin-protein ligase MIB1	−1.57	0.01222727	0.00089299
MRPS27	28S ribosomal protein S27. mitochondrial	−0.99	0.00508971	0.02594273
MRPS6	28S ribosomal protein S6. mitochondrial	−0.97	0.04246351	0.0274775
MYO1C	Unconventional myosin-Ic	0.88	0.01406586	0.01647324
NEGR1	Neuronal growth regulator 1	−0.95	0.03486772	0.03033832
NMT2	Glycylpeptide N-tetradecanoyltransferase 2	−1.41	0.01500637	0.00257116
NOL3	Nucleolar protein 3	−1.16	0.02839415	0.01057664
NTM	Neurotrimin	−1.13	0.00862143	0.01234019
NUMB	Protein numb homolog	−0.90	0.00073514	0.03813309
PACSIN3	Protein kinase C and casein kinase substrate in neurons protein 3	−0.89	0.0372482	0.04071722
PAPOLA	Poly(A) polymerase alpha	−1.39	0.00978221	0.00286055
PCBP2	Poly(rC)-binding protein 2	2.49	0.02675666	1.866E-09
PDE12	2.5-phosphodiesterase 12	−2.19	0.02395318	5.6127E-06

PKN2	Serine/threonine-protein kinase N2	−1.42	0.00967764	0.00234106
PTP4A2;PTP4A1	Protein tyrosine phosphatase type IVA 2;Protein tyrosine phosphatase type IVA 1	1.00	0.03041913	0.00819236
RAB35	Ras-related protein Rab-35	−0.98	0.02901174	0.02632575
RPL22L1	60S ribosomal protein L22-like 1	1.02	0.01187401	0.00719492
RUNX2	Runt-related transcription factor 2	−1.28	0.02650606	0.00547683
SAR1A	GTP-binding protein SAR1a	0.71	0.02283106	0.04095937
SMG8	Protein SMG8	−1.15	0.02471592	0.01160385
TMEM192	Transmembrane protein 192	0.69	0.02382637	0.04621599
TNS3	Tensin-3	1.04	0.00976532	0.00632694
UBAC2	Ubiquitin-associated domain-containing protein 2	1.23	0.04273789	0.00166153
YIPF6	Protein YIPF6	−0.99	0.03002568	0.02601649
YY1;ZFP42	Transcriptional repressor protein YY1;Zinc finger protein 42 homolog	−1.01	0.02415422	0.02322173

Table S7. Proteins showing a significant difference between osteoblast donors above and below 60 years of age. For each protein, the table presents the corresponding gene name, protein name, fold change from the comparison of elderly/younger donors (log_2-transformed), the *p*-value and the z score.

Gene Name	Protein Name	Fold Change Elderly/Young	*p*-value	*z*-score
ACO1	Cytoplasmic aconitate hydratase	−0.92	0.0189653	0.01811366
ALG1	Chitobiosyldiphosphodolichol beta-mannosyltransferase	1.03	0.03518987	0.00296325
ANKFY1	Rabankyrin-5	−0.76	0.01206478	0.04843309
AXL	Tyrosine-protein kinase receptor UFO	−0.88	0.00683968	0.02407376
BRIX1	Ribosome biogenesis protein BRX1 homolog	1.10	0.02451452	0.00177249
CDC27	Cell division cycle protein 27 homolog	−1.31	0.04136941	0.00092621
COL12A1	Collagen alpha-1(XII) chain	1.14	0.03423628	0.00130972
COL1A1	Collagen alpha-1(I) chain	2.01	0.03574206	1.7748E-07
COMMD1	COMM domain-containing protein 1	-0.87	0.02677572	0.02493297
DERA	Deoxyribose-phosphate aldolase	2.36	0.00180329	1.6698E-09
DGKA	Diacylglycerol kinase alpha	−0.84	0.01308061	0.03079946
DNMBP	Dynamin-binding protein	−0.80	0.02436019	0.03800298
DPP4	Dipeptidyl peptidase 4;Dipeptidyl peptidase 4 membrane form	−2.75	0.00556425	2.6305E-12
EDIL3	EGF-like repeat and discoidin I-like domain-containing protein 3	−1.29	0.0120985	0.00110111
EPB41L1	Band 4.1-like protein 1	−1.26	0.00596232	0.00142437
GAR1	H/ACA ribonucleoprotein complex subunit 1	−0.95	0.0294831	0.01517147
GAS6	Growth arrest-specific protein 6	−1.12	0.0119162	0.00442479
GNE	Bifunctional UDP-N-acetylglucosamine 2-epimerase	0.62	0.02649509	0.03857883
GSTM2	Glutathione S-transferase Mu 2	−1.47	0.0354085	0.00021246
HLA-A	HLA class I histocompatibility antigen. A-69 alpha chain	−1.21	0.01623657	0.00216293
L3HYPDH	Trans-3-hydroxy-L-proline dehydratase	−1.01	0.03641763	0.01005164
METTL7A	Methyltransferase-like protein 7A	−1.31	0.03613517	0.00095799
MYCBP2	E3 ubiquitin-protein ligase MYCBP2	−0.85	0.01300965	0.02860529
MYO9B	Unconventional myosin-IXb	−0.97	0.02360738	0.01279615
NUP43	Nucleoporin Nup43	1.01	0.01880347	0.00333926
PPL	Periplakin	−1.98	0.04990482	5.5718E-07
PTPRF	Receptor-type tyrosine-protein phosphatase F	0.63	0.00363919	0.03596013
RPL38	60S ribosomal protein L38	3.31	0.03381556	0
SCRN1	Secernin-1	−1.05	0.03716872	0.00754678

SEC11A	Signal peptidase complex catalytic subunit SEC11A	1.67	0.00994822	8.9979E-06
SH2D4A	SH2 domain-containing protein 4A	−1.21	0.02382157	0.00223084
SLC8A1	Sodium/calcium exchanger 1	1.53	0.03329056	4.0917E-05
STRN	Striatin	−0.79	0.03976304	0.03907754
TIPRL	TIP41-like protein	−0.86	0.0305352	0.02721873
TRADD	Tumor necrosis factor receptor type 1-associated DEATH domain protein	−0.84	0.00406154	0.02943871
UAP1L1	UDP-N-acetylhexosamine pyrophosphorylase-like protein 1	−0.84	0.00522373	0.03060842
UROS	Uroporphyrinogen-III synthase	−0.93	0.03501364	0.01668723
VPS45	Vacuolar protein sorting-associated protein 45	0.61	0.01243271	0.03915107
WDR75	WD repeat-containing protein 75	1.34	0.00861015	0.00024536

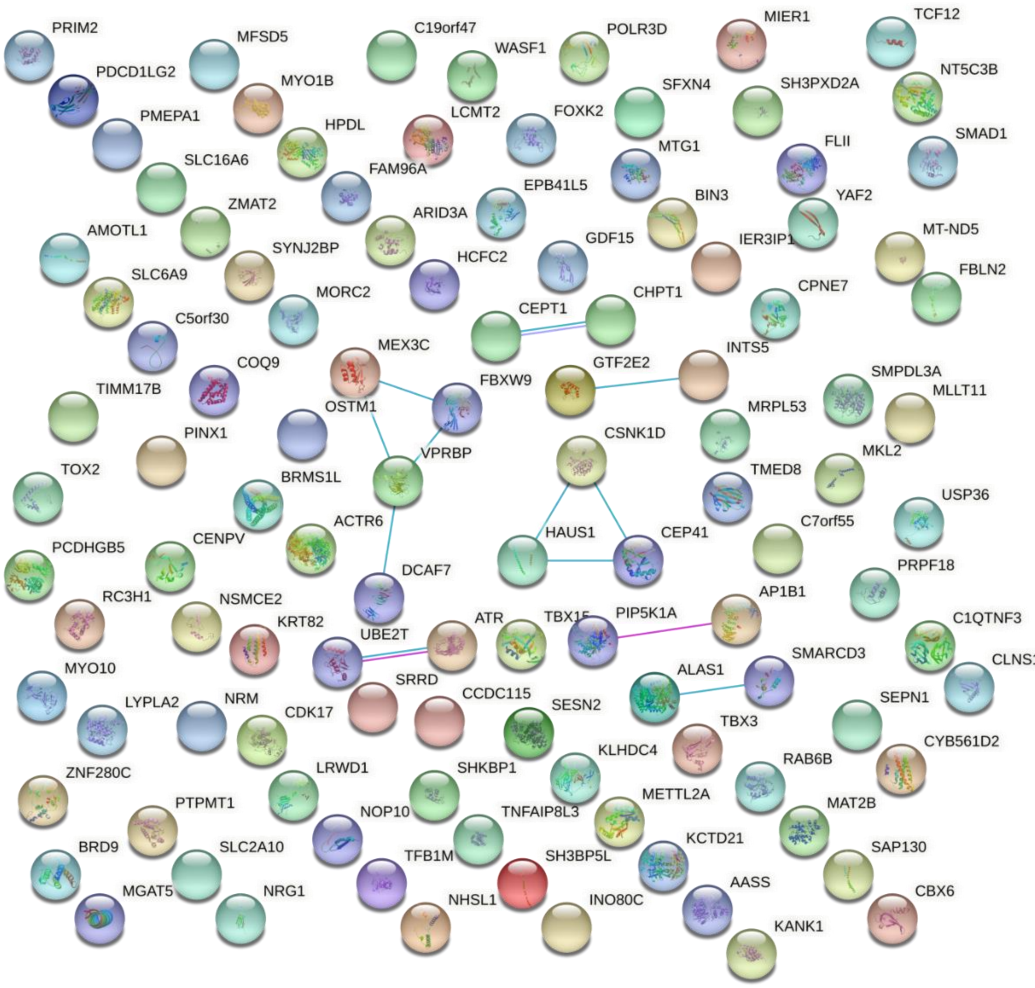

Figure S1. Network analysis of MSC-specific proteins, i.e. proteins only quantified for MSCs but not for any of the osteoblast population. The network was generated by the String database.

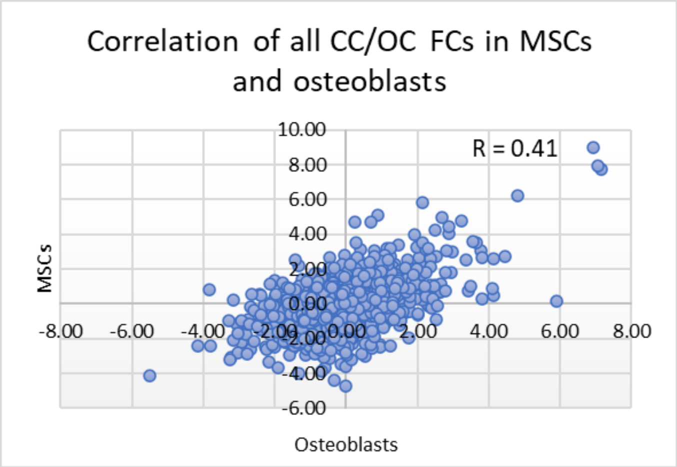

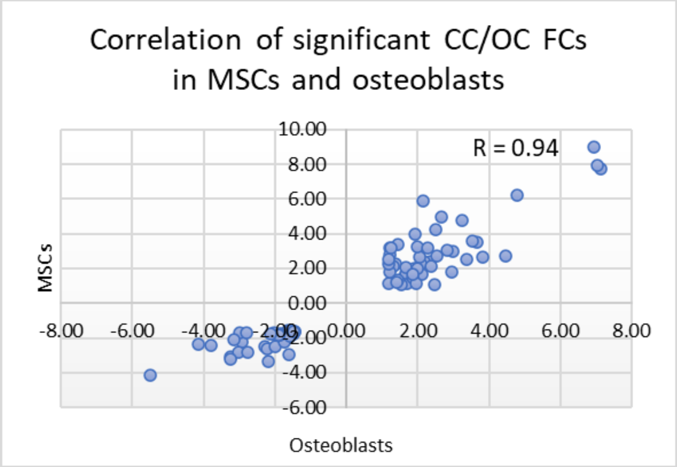

Figure S2. Scatter plot of the fold change (FC) for cells cultured in suboptimal serum-free IMDM medium (CC, cultured cells) vs the original cell (OC); a presentation of the fold changes (FCs) for osteoblasts and MSCs. The upper figure presents the results for 3677 proteins with fold change pairs in at least three donor samples for each cell type, and each dot represents a protein (Pearson R = 0.41). The lower plot presents the results for the 87 proteins which were significantly altered by the suboptimal IMDM culture in both cell types (Pearson R = 0.94).
